# Bioactivities and Phytochemical Profiling of *Trigynaea axilliflora* from Brazil

**DOI:** 10.1021/acsomega.5c08985

**Published:** 2025-11-19

**Authors:** Natyele Rodrigues Fonseca, Diogo Folly Gomes Andrade, Julia Chaves Scaffo, Bismarck Rezende, Vitória Macario de Simas Gonçalves, Yure Bazilio dos Santos, Maria Eduarda Barros de Andrade, Karine Simões Calumbi, Milena Longue, Bruno de Araújo Penna, Adriana Quintella Lobão, Leandro Machado Rocha, Alessandra Leda Valverde, Guilherme Carneiro Montes, Aislan Cristina Rheder Fagundes Pascoal, Lucas Silva Abreu

**Affiliations:** † Instituto de Química, 28110 Universidade Federal Fluminense, Niterói 24020-141, RJ, Brazil; ‡ Faculdade de Farmácia, Universidade Federal Fluminense, Niterói 24241-000, RJ, Brazil; § Departamento de Farmacologia e Psicobiologia, Instituto de Biologia Roberto Alcantara Gomes, 28130 Universidade do Estado do Rio de Janeiro, Rio de Janeiro 20551-030, RJ, Brazil; ∥ Instituto de Saúde de Nova Friburgo, Universidade Federal Fluminense, Nova Friburgo 28625-650, RJ, Brazil; ⊥ Instituto Biomédico, Universidade Federal Fluminense, Niterói 24210-130, RJ, Brazil; # Instituto de Biologia, Departamento de Biologia Geral, Universidade Federal Fluminense, Niterói 24210-201, RJ, Brazil

## Abstract

Brazil is a country with the greatest biodiversity in the world, but much of it is still largely unexplored. *Trigynaea axilliflora* (Annonaceae) is a Brazilian plant species that has not been previously studied. This research aimed to characterize the chemical constituents and evaluate the biological activities of the extract, fractions and isolated compounds. Xylopine, a major compound isolated from this extract, is an aporphine alkaloid and a chemotaxonomic marker of this family, whose structure was confirmed by ^1^H/^13^C NMR and HRESIMS. HPLC-UV-ESI-qTOF dereplication indicated that xylopine and seven other aporphine alkaloids were present in the crude extract. A solvent washing procedure allowed the recovery of 138 mg of xylopine, with a yield of 13.8% (138 mg/g crude extract) and 95% purity by HPLC-DAD. Cytotoxicity by the MTT assay showed that xylopine was active against MG-63 osteosarcoma cells (IC_50_ = 5.5 μg/mL), with a selectivity index (SI = 0.63) indicating a favorable selectivity compared to doxorubicin (SI = 0.22). Antibacterial assays demonstrated the inhibition of *Staphylococcus aureus* and *Escherichia coli* by the ethanolic extract and chloroform fraction (MIC 125–500 μg/mL), as well as by xylopine (MIC 62.5 μg/mL for *S. aureus* and 31.2 μg/mL for *E. coli*). To evaluate antinociceptive activity, the formalin test was used, and both the ethanolic extract and xylopine significantly reduced nociceptive behavior during the inflammatory phase from 123.8 ± 13.7 s (100%) to 71.3 ± 10.2 s (57.5%) and 1.3 ± 0.4 s (1%), respectively, at doses of 30 mg/kg. In silico pharmacokinetic analysis using SwissADME predicted favorable drug-likeness and oral bioavailability for xylopine, supporting its potential as a drug candidate. This study presents the first report on the chemical profile and bioactivities of *T. axilliflora*, suggesting it as a possible natural source of xylopine. This finding highlights the importance of phytochemical and pharmacological research in understanding Brazil’s understudied flora.

## Graphical abstract

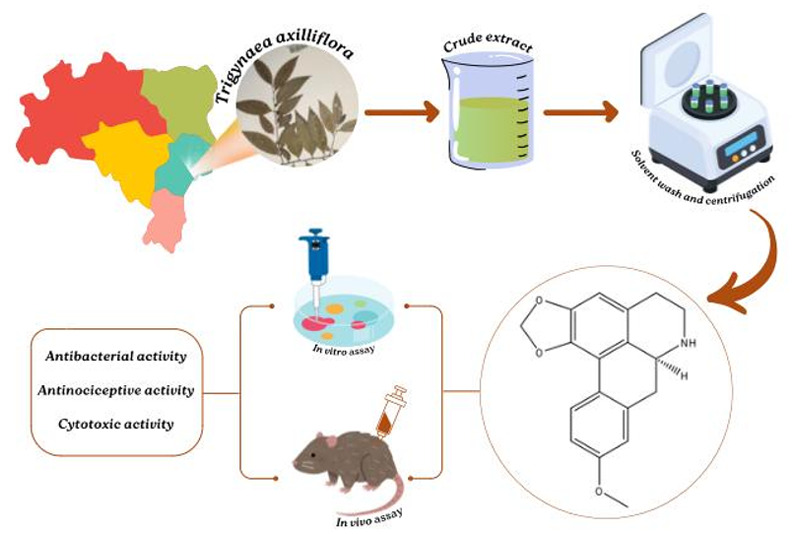

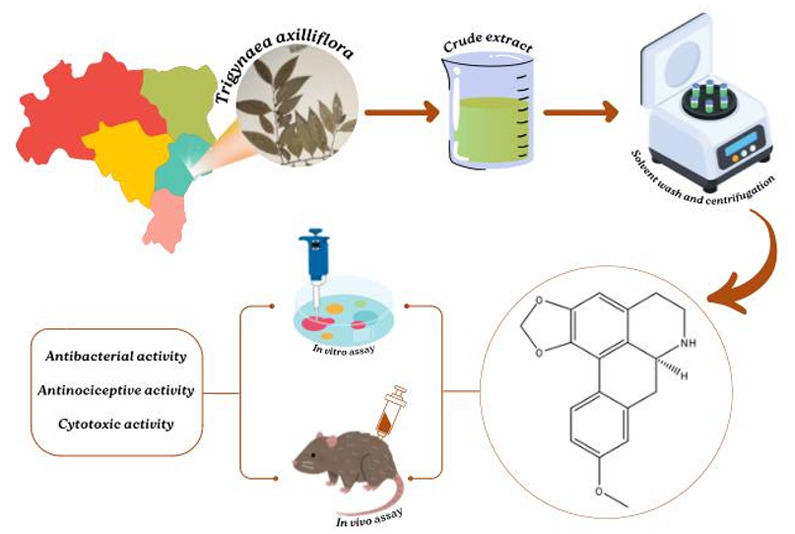

## Introduction

The Amazon and Atlantic Forests in Brazil are hotspots of biodiversity, which host a significant concentration of species from the Annonaceae family.^1,2^ Renowned for their chemical diversity, this family produces a wide array of specialized metabolites, such as flavonoids, coumarins, lactones, phenols, acetogenins, and alkaloids.^3^ Within this context, isoquinoline alkaloids (IA) are particularly noteworthy. Compounds like berberine, palmatine, and magnoflorine demonstrate remarkable polypharmacology, with concurrent anti-inflammatory, antibacterial, anticancer, and neuroprotective effects. The therapeutic versatility of berberine is further highlighted by other reported activities, including antiviral and antidiabetic effects, which are attributed to its anti-inflammatory and antioxidant mechanisms.^4,5^

Aporphines, a subclass of IAs, have received increasing attention due to their structural diversity and broad spectrum of biological activities. In 2022, the rare *N*-benzyl isoquinoline alkaloid 3,4–2*H*-tomentelline was isolated from *Corydalis tomentella* and exhibited significant cytotoxicity against HepG2 cells.^6^ Numerous biological activities attributed to aporphines, such as cardioprotective, antiulcer, and analgesic effects, demonstrate the therapeutic potential of this class.^6^ Xylopine, a member of this subclass, has been reported to be from diverse species within this family, serving as a chemotaxonomic marker for this group. Several biological activities, including antifungal, cytotoxic, and antileishmanial properties, have been described for this compound.^7–9^ However, only in vitro activities were reported, possibly due to the low isolated amounts of this compound.

Although several genera of the Annonaceae family have been the subject of chemical and pharmacological studies, some, such as *Trigynaea*, have not yet been reported in the literature. An exception is a preliminary study by Pompilho et al. (2014), which was based on phytochemical screening, indicating the presence of alkaloids, anthraquinones, coumarins, tannins, and saponins in the methanolic extract of the leaves of *T. oblongifolia*, as well as antioxidant activity and toxicity against *Artemia salina*.^10^ This genus has only nine described species, three of which are endemic to southeastern of Brazil.^11^ Among these, *Trigynaea axilliflora*, found in the state of Rio de Janeiro, has no descriptions of its chemical and biological profile. Thus, this study aims to investigate the bioactivities and phytochemical profiling of *T. axilliflora* from Brazil, reporting the isolation of xylopine and evaluating the cytotoxic, antimicrobial, and antinociceptive potential.

## Results and Discussion

### Isolation of Xylopine from *T. axilliflora* Leaves

The ethanolic crude extract (22.9 g) from the leaves of *T. axilliflora* was partitioned with *n*-hexane, CHCl_3_, EtOAc, and BuOH. The CHCl_3_ phase (5.94 g) was fractionated by Vacuum Liquid Chromatography (VLC), affording 14 fractions (C1–C14). Fraction C8 presented a dark amorphous solid that was washed with small amounts of CHCl_3_, resulting in purified xylopine (105 mg, 0.30% yield relative to the extract; Figure 1). The structure was determined by ^1^H and ^13^C NMR spectroscopy (Figures S1–S5, Supporting Information), with data consistent with those previously reported for this aporphine alkaloid.^8,12^ Attempts to isolate other compounds by conventional chromatographic methods resulted in repeated recovery of xylopine, suggesting that this compound is the predominant alkaloid in this fraction, probably in the extract.

**Figure 1:**
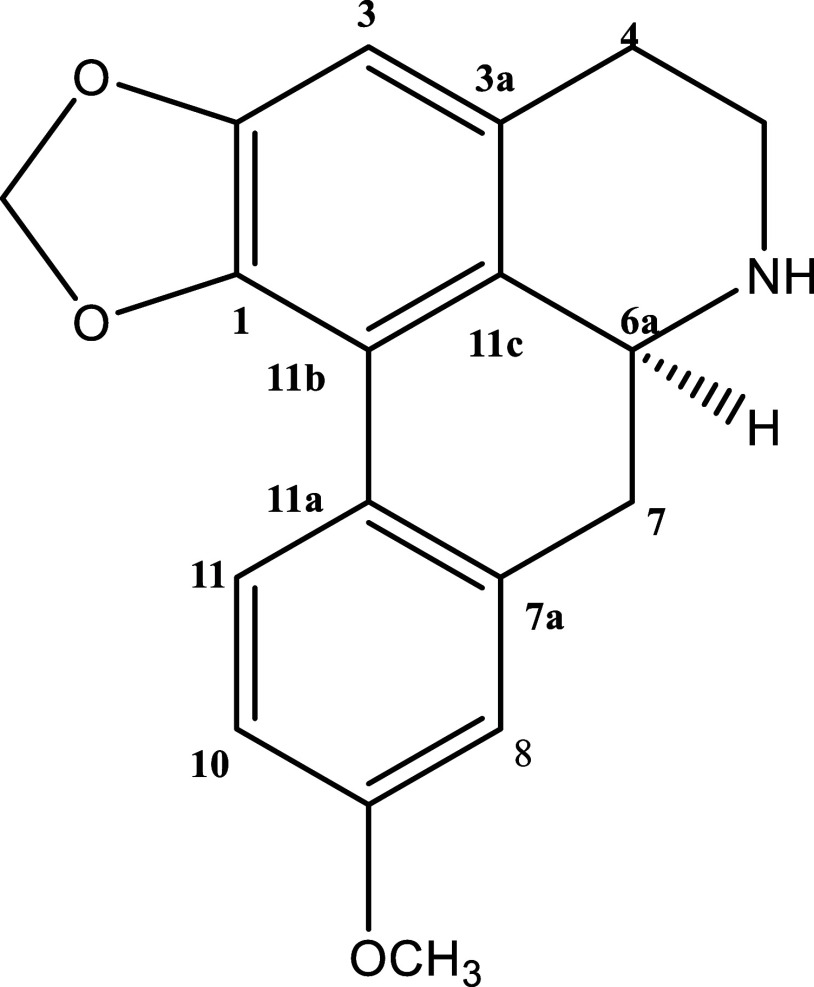
Structure of xylopine.

To confirm this observation, the crude ethanolic extract was analyzed by HPLC-UV-ESI-qTOF, which revealed a chromatographic profile with the main compound corresponding to xylopine (Figure 2). A dereplication study was conducted to determine the chemical composition of the extract, resulting in the annotation of 8 aporphine alkaloids (Table S1 in the Supporting Information).

**Figure 2:**
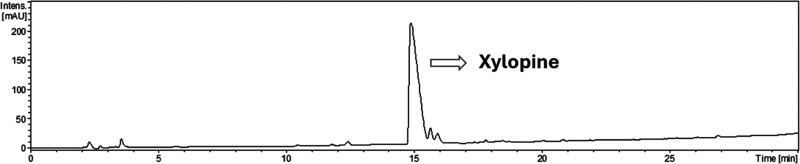
Chromatogram at 254 nm of ethanolic crude extract from leaves of *Trigynaea axilliflora* by HPLC-UV.

Given the dominance of xylopine in *T. axilliflora*, large-scale purification strategies were developed based on differential solubility and acid–base extraction to yield an alkaloid-rich fraction. The most effective method (1) involved sequential washing of ethanolic crude extract with hexane followed by acetone, yielding a precipitate containing 138 mg of xylopine per gram of extract (138 mg/g crude extract, 95,5% of purity). Washing with only acetone (2) resulted in a precipitate with a slightly lower yield (109 mg/g crude extract, 91,8% of purity). In contrast, acid–base extraction (3) yielded an alkaloid-rich fraction with higher impurity level (431 mg/g crude extract, 55,9% of purity), being inefficient in providing purified xylopine, as indicated by ^1^H NMR and HPLC-DAD analysis. Furthermore, the crude extract was analyzed in the same conditions indicating the presence of 36,7% of xylopine. The results are presented in Table 1, and corresponding spectra and chromatograms from the purifications can be found in the Supporting Information (Figures S7–S13).

**Table 1:** Yield and Purity of Xylopine Obtained from Purification Strategies

	method 1	method 2	method 3
weight of purified xylopine (mg)^a^	138	109.9	431^b^
yield (%)	13.8	10.9	43.1
purity (%)^c^	95,5	91,8	55,9

a Weight of purified xylopine (mg) per 1.0 g of crude extract.

b The low purity of xylopine indicates that it is unpurified.

c *The purity of xylopine was evaluated by HPLC-DAD analysis*.

Finally, 10.0 g of ethanolic extract was subjected to the optimized solvent-washing procedure (method 1), yielding 1.4 g of purified xylopine to be sent to in vitro and in vivo biological assays. This predominance contrasts with what is commonly reported in literature based on the isolation of isoquinoline alkaloids. For example, from *Xylopia laevigata*, xylopine (13.4 mg) was isolated, together with 18 other alkaloids with amounts ranging from 1.5 to 21.9 mg.^8^ The high proportion of this single aporphine alkaloid, combined with the absence of significant competing alkaloids, not only simplifies large-scale isolation but also underscores the phytochemical singularity of *T. axilliflora* in comparison to other members of Annonaceae. Furthermore, the presence of xylopine supports its status as a chemotaxonomic marker of this family.^8^

### Biological In Vitro Assay

#### Cytotoxic Evaluation

The cell viability of MG-63 (osteosarcoma) cells and HaCat (nontumorigenic keratinocyte cells) after sample exposure was assessed using the MTT assay, which measures metabolic activity.^13^ These assays allowed for the determination of the concentration required to inhibit 50% of cell viability (IC_50_). Both cell lines were treated with Negative Control (CTRL), Positive Control (doxorubicin), TAFE–E (crude extract); TAFE–H (hexane fraction), TAFE–C (chloroform fraction), TAFE–A (ethyl acetate fraction), TAFE–B (butanol fraction) (Figure 3 and Table 2).

**Figure 3:**
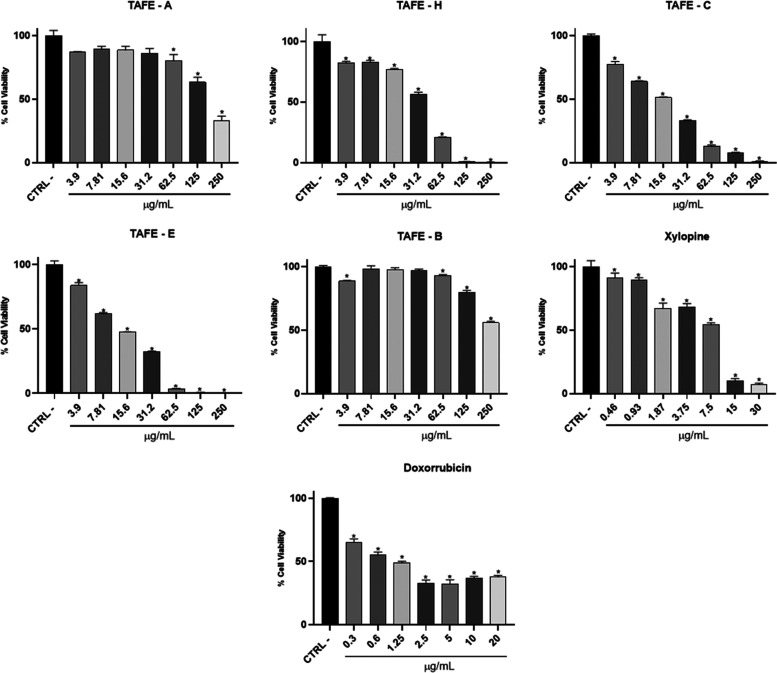
Results of the MG-63 cell viability assay using the MTT method, in percentage (%), through the bar graph, comparing cells of the MG-63 cell line, after receiving the respective treatments: TAFE–E (crude extract); TAFE–H (hexane fraction), TAFE–C (chloroform fraction), TAFE–A: (ethyl acetate fraction); TAFE–B: butanol fraction, xylopine and doxorubicin (positive control). One-way ANOVA was used for statistical analyses, followed by Dunnett’s test, considering the control group (CTRL) with 100% cell viability. * Indicates the sample concentration at which there was a statistically significant difference (*p* < 0.05) when compared to the control (CTRL).

**Table 2:** IC_50_ Values and Selectivity Index (SI) of the Tested Samples, as Well as of the Positive Controls Doxorubicin, in MG-63 and Non-tumor HaCat Cells^a^

samples	IC_50_ MG-63 (μg/mL)	IC_50_ HaCat (μg/mL)	SI
TAFE-E	13.28	15.23	1.14
TAFE-H	30.18	36.06	1.19
TAFE-C	14.17	7.30	0.51
TAFE-A	17.60	147.20	8.36
TAFE-B	291.70	337.80	1.15
XYLOPINE	5.50 (18.6 μM)	3.49 (11.82 μM)	0.63
DOXORUBICIN	1.34 (2.47 μM)	0.29 (0.55 μM)	0.22

a TAFE–E (crude extract); TAFE–H (hexane fraction), TAFE–C (chloroform fraction), TAFE–A: (ethyl acetate fraction); TAFE–B: butanol fraction.

In osteosarcoma cells (MG-63), it was statistically evident that cell metabolism was inhibited with all treatments, except TAFE-B, when compared to the negative control (100% of viability). The TAFE-E showed a statistically significant difference compared with the negative control sine at a dose of 3.9 μg/mL, with an IC_50_ of 13.28 μg/mL, demonstrating high activity. The TAFE-H sample showed a statistically significant difference compared with the negative control in doses, starting at 3.9 μg/mL, with an IC_50_ of 30.18 μg/mL. In TAFE-C, a reduction in cell viability was observed at all concentrations, with a statistically significant difference compared to the negative control, yielding an IC_50_ of 14.17 μg/mL. In TAFE-A, IC_50_ was 17.6 μg/mL. The isolated compound xylopine had an IC_50_ value of 5.5 μg/mL, and the positive control, both doxorubicin samples, showed a concentration of 1.34 μg/mL.

The HaCat, a nontumor cell line, demonstrated that TAFE-B did not alter cell metabolism at any concentration tested. As a positive control, doxorubicin inhibited the metabolic activity of nontumor cells at all concentrations tested, with an IC_50_ of 0.2989 μg/mL.

TAFE-E exhibited cytotoxic effects at concentrations above 7.81 μg/mL, with an IC_50_ of 15.23 μg/mL. TAFA-H, at 3.9 μg/mL, did not alter cell viability, showing an IC_50_ of 36.66 μg/mL. For TAFE-C, all tested concentrations significantly reduced cell viability compared with the negative control, with an IC_50_ of 7.3 μg/mL. The TAFE-A sample showed a decrease in cell viability only at higher concentrations, with an IC_50_ of 147.2 μg/mL. TAFE-B presented statistically significant differences relative to the negative control only at 125 and 250 μg/mL. Xylopine reduced cell viability at a concentration of 1.87 μg/mL, with an IC_50_ of 3.492 μg/mL. Doxorubicin, while exhibiting the lowest IC_50_ (0.2989 μg/mL), also inhibited metabolic activity in nontumor cells at all tested concentrations (Figure 4).

**Figure 4:**
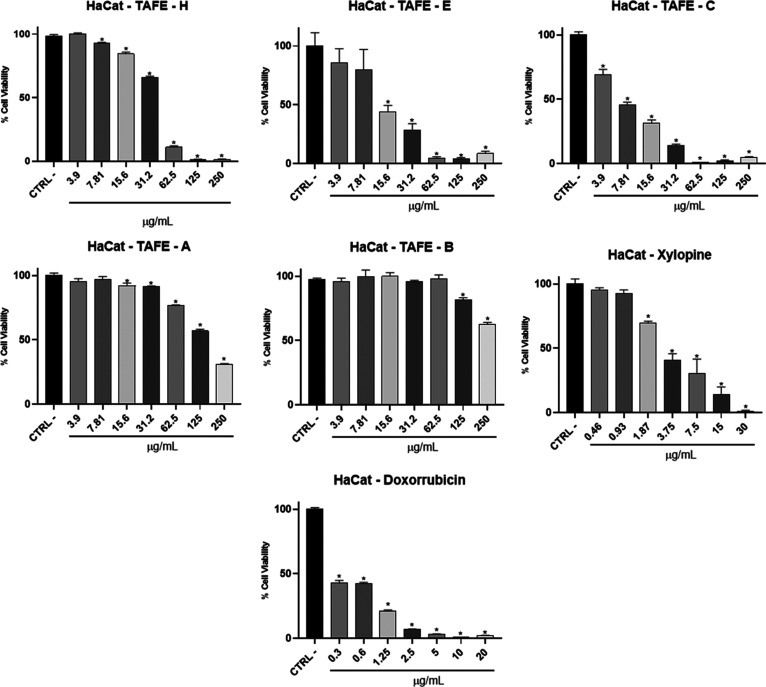
Results of the HaCat cell viability assay using the MTT method, in percentage (%), through the bar graph, comparing cells of the HaCat cell line, after receiving the respective treatments: TAFE–E (crude extract); TAFE–H (hexane fraction), TAFE–C (chloroform fraction), TAFE–A: (ethyl acetate fraction); TAFE–B: butanol fraction, xylopine and doxorubicin (positive control). One-way ANOVA was used for statistical analysis, followed by Dunnett’s test, with the control group (CTRL) considered to have 100% cell viability. “*” indicates the sample concentration at which there was a statistically significant difference (*p* < 0.05) when compared to the control (CTRL).

Although both xylopine and doxorubicin reduced the viability of nontumor HaCat cells, the vigorous cytotoxic activity of xylopine against MG-63 osteosarcoma cells, combined with its higher IC_50_ in nontumor cells compared to doxorubicin, highlights its potential as a promising anticancer lead compound. Importantly, given the growing clinical challenge of multidrug resistance (MDR), particularly in osteosarcomas and other aggressive tumors, the identification of compounds like xylopine that may act through mechanisms distinct from conventional chemotherapeutics is of high translational value. These findings suggest that, despite some cytotoxicity to healthy cells, xylopine warrants further investigation. Future studies should focus on structural optimization, elucidation of its mechanism of action, including potential effects on MDR pathways, and the development of targeted delivery strategies to enhance selectivity and minimize off-target effects.

The results obtained in this study are consistent with previous reports describing the cytotoxic activity of aporphine alkaloids such as xylopine. The cytotoxicity of xylopine was evaluated in eight human cancer cell lines (MCF7, HCT116, HepG2, SCC-9, HSC-3, HL-60, K-562, and B16–F10) and in two nontumor cell types (MRC-5 and PBMC) using the Alamar Blue assay after 72 h of incubation. Xylopine exhibited IC_50_ values ranging from 6.4 μM in HCT116 cells to 26.6 μM in SCC-9 cells. Analysis of cell cycle distribution in HCT116 cells by flow cytometry after 24 and 48 h of exposure revealed that xylopine treatment significantly increased the proportion of cells arrested in the G2/M phase compared with the control group (30.7% in control vs 57.2, 58.5, and 54.0% at 3.5, 7, and 14 μM, respectively, after 24 h; and 23.8% in control vs 52.0, 52.7, and 40.8% at the same concentrations after 48 h). This G2/M phase arrest was accompanied by an increase in internucleosomal DNA fragmentation, indicating the induction of apoptosis. The possible course of action is to investigate the effect of xylopine on intracellular reactive oxygen/nitrogen species (ROS/RNS) levels in HCT116 cells using flow cytometry.^14^ Taken together, these findings support the antitumor potential of xylopine and underscore the need for in vivo studies to further evaluate its systemic effects and therapeutic relevance.

#### Antibacterial Activity

The extracts from *T. axilliflora* exhibited antibacterial properties against *Staphylococcus aureus* and *Escherichia coli*, with MIC values of 125 μg/mL for both strains with TAFE–E, and 125 μg/mL for *S. aureus* and 500 μg/mL for *E. coli* with TAFE–C. However, the MBC values were higher than the MIC values, ranging from 500 μg/mL for *S. aureus* to 2000 μg/mL for *E. coli* treated with TAFE–C, while with TAFE–E, the MBC values were 250 μg/mL for both strains (Table 3). Statistical analysis revealed significant differences in MIC values between the extracts and vancomycin for *S. aureus*, and between TAFE–C and the control for *E. coli*. Regarding MBC, only TAFE–C showed a significant difference compared with both controls, including xylopine for *E. coli*.

**Table 3:** Minimum Inhibitory Concentration (MIC) and Minimum Bactericidal Concentration (MBC) of *T. axilliflora* Extracts and Xylopine Against Bacterial strains^a^ ^,^ ^b^

	bacterial strains			
samples	*S. aureus* ATCC 29213		*E. coli* ATCC 25922	
	MIC (μg/mL)	MBC (μg/mL)	MIC (μg/mL)	MBC (μg/mL)
TAFE–E	125*	250	125	250
TAFE–C	125*	500**	500**	2000*
xylopine	62.5	250	31.2	>2000**
vancomycin	1	1	-	-
ciprofloxacin	-	-	<3.9	<3.9

a Statistical analyses were performed using one-way ANOVA with Dunnets post-test. *P* < 0.05; **P < 0.005.

b TAFE–E (Crude extract); TAFE–C (chloroform fraction).

The present data expand the understanding of antibacterial activity within the Annonaceae family. While species such as *Annona muricata* have been extensively investigated, the biological activity of *T. axilliflora* remains limited. This scarcity of previous research enhances the relevance of our findings, which provide new insights into the antimicrobial potential of this understudied species. For instance, Dey et al. (2025) reported the inhibitory activity of ethanolic (1200 μg/mL), ethyl acetate (120 μg/mL), chloroform (75 μg/mL), and *n*-hexane (550 μg/mL) of *A. muricata* bark against *E. coli* *.* ^15^ In addition, Pinto et al. (2017) demonstrated that the methanolic extract of *A. muricata* leaves presented MIC/MBC values of 156/156 μg/mL against *S. aureus* and 625/2500 μg/mL against *E. coli*.^16^

In addition to *A. muricata*, numerous other species within the Annonaceae family have exhibited significant antibacterial potential. Extracts of *Annona cherimola*, *Annona glabra*, *Annona reticulata*, *Rollinia mucosa*, and *Annona montana* exhibited growth inhibition ranging from 10% to 80% against *S. aureus*, *Enterococcus faecalis*, *Bacillus subtilis*, *E. coli*, and *Pseudomonas aeruginosa*.^17^ Extracts from the roots and bark of *Anonidium mannii* also inhibited Gram-negative strains, including *E. coli*, with MIC values between 256 and 1024 μg/mL and MBC values ≥1024 μg/mL.^18^ Likewise, *Annickia chlorantha* displayed inhibitory activity against *S. aureus* at 1000 μg/mL, whereas *Annona hypoglauca* showed no inhibition (MIC >100 μg/mL) against *S. aureus* and *E. coli*.^19,20^ Collectively, these reports emphasize the broad antimicrobial potential distributed among members of the Annonaceae family and support that the plant evaluated in the present study exhibits comparable or even superior inhibitory activity against *S. aureus* and *E. coli*.

In contrast to the crude extract, xylopine exhibited lower inhibitory activity against both bacterial strains, with MIC values ranging from 62.5 to 31.2 μg/mL and MBC values exceeding 2000 μg/mL for *S. aureus* and 250 μg/mL for *E. coli*, respectively (Table 3). A study by Nugraha et al. (2019) identified that an alkaloid fraction from the methanolic extract of *A. muricata* roots has antimicrobial activity above 32 μg/mL against both *S. aureus* and *E. coli*.^21^ However, the present study identified the activity at 62.5 μg/mL for *S. aureus*. The variation in inhibition observed among the *E. coli* samples may be due to bioactive compounds present in other extract fractions, as activity was also detected in the ethanolic and chloroform fractions. Furthermore, aporphine alkaloids have been reported to possess antibacterial activity, including inhibition of *S. aureus* at 62.5 μg/mL, as demonstrated for alkaloid anonaine.^22^ Therefore, the results obtained suggest that the antibacterial activity of *T. axilliflora* extracts may be associated with the aporphine alkaloid, xylopine.

### Biological In Vivo Assay

#### Antinociceptive Activity

The formalin-induced nociception model revealed that intraperitoneal injection of TAFE (Figure 5) and xylopine (Figure 6) produced significant antinociceptive effects. In the neurogenic phase, licking time in the morphine-treated group was reduced to 20.2 ± 5.3 s (35.6%) compared with 56.6 ± 4.9 s (100%) in the vehicle group (Figure 5A). During the inflammatory phase, animals treated with TAFE exhibited a reduction in licking time from 123.8 ± 13.1 s (100% to vehicle) to 70.8 ± 11.0 (57%), 31.4 ± 14.0 (25%), and 71.3 ± 10.2 s (57.5%) at the doses of 10, 30, and 100 mg/kg, respectively. The groups treated with morphine and ASA showed reductions to 13.7 ± 7.1 (11%) s and 49.7 ± 4.7 (40%) s, respectively (Figure 5B).

**Figure 5:**
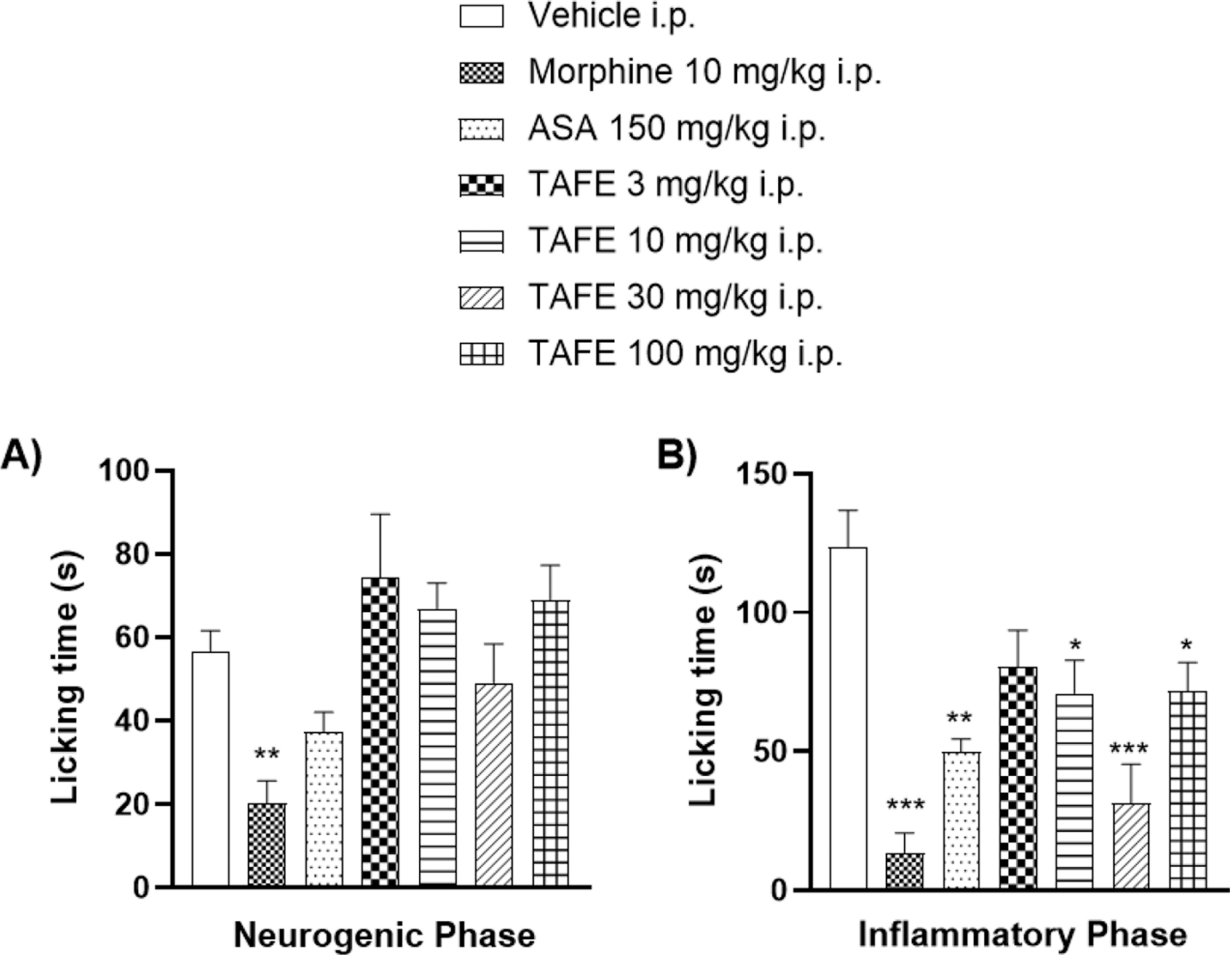
Effects of intraperitoneal administration of vehicle, morphine (30 mg/kg), ASA (300 mg/kg), or TAFE (3–100 mg/kg) on the neurogenic (A) and inflammatory (B) phases of the formalin test. Bars represent mean ± SEM (*n* = 7). *p < 0.05, **p < 0.01, and *p < 0.001 vs vehicle group. Data were analyzed by one-way ANOVA followed by Dunnett’s post hoc test.

**Figure 6:**
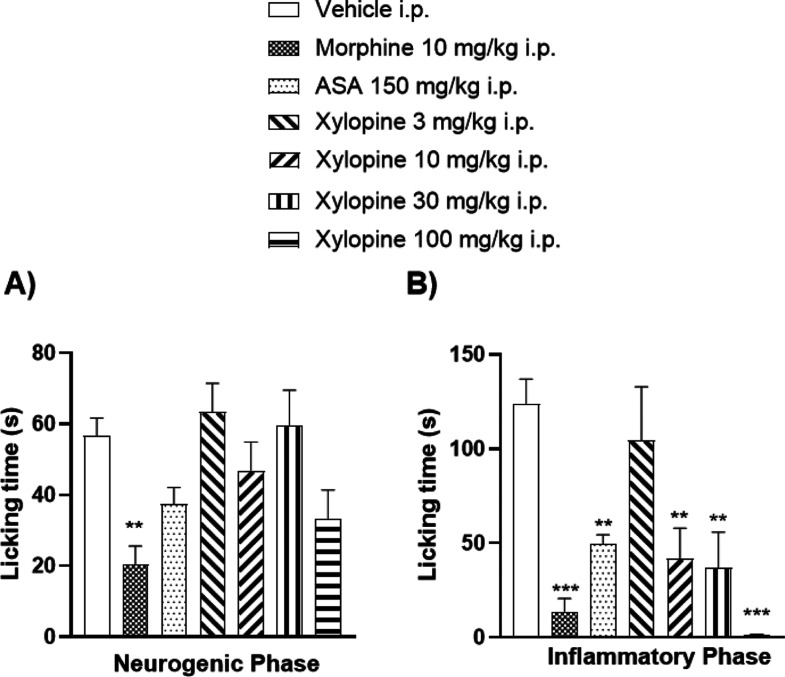
Effects of intraperitoneal administration of vehicle, morphine (30 mg/kg), ASA (300 mg/kg), or xylopine (3–100 mg/kg) on the neurogenic (A) and inflammatory (B) phases of the formalin test. Bars represent mean ± SEM (*n* = 7). *p < 0.05, **p < 0.01, and *p < 0.001 vs vehicle group. Data were analyzed by one-way ANOVA followed by Dunnett’s post hoc test.

In the neurogenic phase, xylopine did not show antinociceptive activity, with only the morphine-treated group exhibiting a significant effect. However, during the inflammatory phase, animals treated with xylopine at doses of 10, 30, and 100 mg/kg (i.p.) showed a reduction in licking time to 41.7 ± 16.1 (33.6%), 37.0 ± 18.8 (29.8%), and 1.3 ± 0.4 s (1%), respectively, compared with 123.8 ± 13.1 s (100%) in the vehicle group.

Our findings demonstrate that the ethanolic extract of TAFE produces robust antinociceptive effects in an acute pain model. When the principal constituent isolated from the extract, xylopine, was evaluated, a more pronounced antinociceptive effect was observed in the inflammatory phase compared with the ethanolic extract of TAFE. Mori et al. (2011) reported that *N*-acetylxylopine (AXyl), a stable derivative of xylopine, exhibits analgesic properties against inflammatory pain by acting at peripheral sites, which corroborates our results.^23^ Although previous reports on xylopine mainly described cytotoxic, and antileishmanial activities, its structural classification as an isoquinoline alkaloid provided a strong rationale for evaluating potential analgesic effects, since this class is well-known to include compounds with analgesic and anti-inflammatory properties, such as morphine and magnoflorine, respectively.^24^ Therefore, our study represents the first direct evidence of the antinociceptive activity of xylopine. Although these findings provide important insights into the antinociceptive effects of TAFE and xylopine in models of acute pain, further studies are required to elucidate the mechanisms underlying the antinociceptive action of xylopine in acute settings, as well as to evaluate its potential efficacy in animal models of chronic pain. Such investigations will be crucial in determining whether xylopine acts through the modulation of specific nociceptive pathways, cannabinoid or opioid receptor systems, or through anti-inflammatory mechanisms involving cytokines and other mediators. Exploring these aspects could establish xylopine as a promising candidate for the development of new analgesic therapies.

### In Silico Evaluation

#### Computational Prediction of Physicochemical Descriptors (SwissADME)

The in silico analysis of the compound, conducted through the SwissADME platform, revealed a highly favorable pharmacokinetic profile for development as an oral drug candidate. The physicochemical parameters, including molecular weight (295.33 g/mol), a reduced number of hydrogen bond donors and acceptors (HBD = 1; HBA = 4), and polar surface area (TPSA = 39.72 Å^2^), indicate excellent membrane permeability (Table S3). The LogP value (consensus = 2.88) suggests moderate lipophilicity, sufficient to cross biological membranes without compromising solubility. This characteristic, combined with the predicted high gastrointestinal absorption, reinforces the potential for oral administration (tables S4 and S5).

The predictive profile also indicated permeability across the blood–brain barrier (BBB), which broadens the possibilities for applications in the central nervous system (CNS). However, such a property must be carefully evaluated concerning unwanted adverse effects. On the other hand, the compound was identified as a substrate of *P*-glycoprotein (*P*-gp), an efflux protein associated with multidrug resistance (MDR) in tumor cells. Such a characteristic may influence intracellular bioavailability and reduce antiproliferative efficacy in cell lines with high expression of this efflux pump (Table S6).

The analysis of the predicted interactions with isoforms of cytochrome P450 revealed inhibition of CYP1A2, CYP2C19, CYP2D6, and CYP3A4, suggesting a potential risk of drug interactions, especially in oncology polytherapy regimens. However, the absence of structural alerts for PAINS and Brenk, coupled with compliance with the rules of Lipinski, Ghose, Veber, Egan, and Muegge, indicates high “drug-likeness” and viability for optimization (Table S7).

The biological activities observed for *T. axilliflora* and its major alkaloid xylopine are reinforced by the in silico pharmacokinetic predictions. The strong cytotoxic, antibacterial, and antinociceptive effects demonstrated experimentally are consistent with the favorable oral bioavailability, membrane permeability, and predicted gastrointestinal absorption of xylopine revealed by SwissADME. Furthermore, the ability to cross the BBB expands its therapeutic potential, although it raises concerns about possible CNS-related side effects. Notably, while the compound showed promising antiproliferative activity in vitro, its identification as a *P*-gp substrate suggests that intracellular accumulation may be limited in resistant tumor phenotypes, potentially reducing efficacy. This aspect, together with the predicted inhibition of major CYP450 isoforms, underscores the need for careful assessment of pharmacokinetic interactions in future studies. Nonetheless, the alignment between the potent bioactivity of xylopine and its favorable drug-likeness profile underscores its relevance as a promising lead for phytomedicine development, supporting continued investigation into both its therapeutic applications and pharmacological limitations.

Thus, the obtained data support the potential of the compound as a prototype candidate for the development of new phytomedicine, while highlighting the importance of evaluating, in subsequent studies, the impacts of *P*-gp and interactions with CYP450 on therapeutic efficacy.

## Conclusions

This work presents the first report of the bioactivities and chemical profiling of *T. axilliflora*. Xylopine was identified as the main aporphine alkaloid in the species, confirmed by dereplication in HPLC-UV-ESI-qTOF and NMR. The scalable isolation procedure indicates *T. axilliflora* as a natural source of xylopine with diverse biological activities. An in vitro assay of the MG-63 osteosarcoma cell line shows that the xylopine has an SI of 0.63; superior to that of SI from Doxorubicin, 0.22. Xylopine exhibits antibacterial activity, inhibiting the growth of *S. aureus* (62.5 μg/mL) and *E. coli* (31.2 μg/mL). In vivo, the extract from *T. axilliflora* and xylopine reduced nociceptive behavior by 57.5% and 1%, respectively, during the inflammatory phase of the formalin test at the highest dose. This study demonstrated that *T. axilliflora* is an important species from Atlantic Forest, with chemical and biological properties, and future investigation needs to be conducted. Thus, this study emphasizes the importance of ongoing phytochemical research into species that have not yet been investigated, highlighting the rich biodiversity of the Brazilian flora.

## Experimental Section

### General Materials

The solvents used were analytical-grade BIOSCIE and LABSYNTH. Commercial silica gel plates (Whatman) were used in the TLC, layered with a thickness of 0.25 mm on aluminum support (20 × 20 cm). The substances were analyzed using ultraviolet radiation at wavelengths of 254 and 365 nm, as well as iodine vapors. ^1^H and ^13^C Nuclear Magnetic Resonance (NMR) spectra were obtained using BRUKER spectrometers operating at 500 and 125 MHz for hydrogen and carbon, respectively. The solvents used to obtain the spectra were CIL methanol and Aldrich Gold Label tubes (3 and 5 mm). The chromatograms obtained by High Performance Liquid Chromatography (HPLC) are from Shimadzu.

### Plant Material

The aerial parts of *T. axilliflora* (approximately 1.2 kg) were collected in Tijuca National Park (RJ), 22′57′58′S - 43′14′23′W, with authorization from ICMBio (n° 84009) and registration in SISGEN (A01BF82). Prof. Adriana Quintella Lobão identified the species, and the material was deposited in the Niterói herbarium (NIT 11877). After collecting, the material was dried in a ventilated oven at 35 °C for 72 h.

### Extraction and Isolation Procedures

The dried and ground leaves (386.6 g) were extracted with 96% ethanol (three times for 72 h) and concentrated under reduced pressure at 40 °C to obtain the crude extract (37.7 g) with an approximate yield of 10% (w/w). 22.9 g of the extract was resuspended in a solution of MeOH–H_2_O (7:3) and partitioned successively with *n*-hexane (1.85 g), CHCl_3_ (8.69 g), EtOAc (1.21 g), and BuOH (2.11 g), resulting in these phases. After thin-layer chromatography (TLC) analysis, the chloroform fraction (5.94 g) was fractionated by VLC, using unitary and binary mobile phases of CHCl_3_ and MeOH, with increasing polarity. Among the 14 resulting fractions, fraction 8 (93:7) exhibited crystal formation, which was subsequently purified by washing with CHCl_3_ and centrifugation for 5 min at 3000 rpm. After repeatedly removing the supernatant until a color change was observed, the solid (105 mg) was sent for ^1^H NMR analysis. To evaluate the purity of the substance, High Performance Liquid Chromatography (HPLC) analysis was performed using an exploratory gradient, with the 5–100% B method over 35 min, employing 0.1% formic acid in A and MeOH in B. Following the isolation of the compound, methods for its large-scale production were established. The crude extract was then analyzed by HPLC, producing a chromatogram with a significant peak at the same retention time as xylopine. Thus, aliquots of ethanolic crude extract (1.0 g) were used as the starting material and subjected to three methodologies. The first step involved removing fatty acids and other impurities, which was achieved through three washes with hexane under agitation (5 mL for 5 min each). The supernatant was removed, and 15 mL of acetone was added under agitation for 10 min. The mixture was then cooled until the precipitate decanted. Finally, the sample underwent successive centrifugations and was washed with the same solvent. The precipitate was sent for ^1^H NMR and HPLC analysis to compare it with the initial compound. A similar form, the second methodology used only acetone (5 mL for 5 min each), followed by freezing and precipitation with centrifugation. The last was used for acid–base extraction. The crude extract was solubilized with 20 mL of CHCl_3_ and partitioned three times with 10 mL of 3% HCl (v/v). The acid solution was then basified with NH_4_OH and partitioned again with CHCl_3_ to obtain an alkaloid-rich fraction.

### HPLC-UV-ESI-qTOF Analysis

Samples (1.0 mg) were dissolved in 1.0 mL in methanol, centrifuged for 10 min, and subsequently injected into the liquid chromatography system. The analysis was conducted using a high-performance liquid chromatography (HPLC) system (Shimadzu, Kyoto, Japan) coupled to a microOTOF-Q II mass spectrometer (Bruker Daltonics, Billerica, MA, USA) equipped with an electrospray ionization (ESI) source. Chromatographic separation was performed using a Kromasil C18 analytical column (250 mm × 4.6 mm, 5 μm; Shimadzu, Kyoto, Japan). Sample injections (20 μL) were carried out using an autosampler. The mobile phase consisted of 0.1% formic acid in water (solvent A) and methanol (solvent B). The elution gradient was as follows: 0.0–20.0 min (50–100% B); 20.0–26.0 min (100% B); 26.0–27.0 min (100–50% B) and 27.0–30.0 min (50% B). The flow rate was maintained at 0.6 mL/min. The temperature of the column was 40 °C. Instrumental parameters were set as follows: capillary voltage of 4.5 kV, ESI operated in positive ionization mode, end plate offset at 500 V, nebulizer pressure at 40 psi, dry gas (N_2_) flow at 8 mL/min, and a temperature of 200 °C. Collision-induced dissociation (CID) was performed in auto MS/MS mode. Mass spectra were acquired over the *m*/*z* range of 100–1200.

### HPLC-DAD

The samples obtained from the purification methods described above and the crude extract was analyzed using a Shimadzu Prominence chromatograph equipped with a solvent pump: LC-20AT, a self-injector: SIL-20A, a degassing system: DGU-20A, an array detector of diodes: SPD-M20A, one oven: CTO-20A and one system controller: CBM-20A. The column used was a Kromasil C18 (250 mm × 4.6 mm ID, 5.0 μm) and a Kromasil C18 precolumn (4.6 mm ID × 3.0 mm, 5.0 μm). The analysis of the data obtained using HPLC-DAD was carried out with Lab Solutions software (Shimadzu). Samples were filtered on 0.22 μm nylon membranes (Chromastore). The mobile phase consisted of 0.1% formic acid in water (solvent A) and methanol (solvent B). The elution gradient was as follows: 0.0–35.0 min (20–100% B); 35.0–45.0 min (100% B); 45.0–47.0 min (100–20% B) and 47.0–60.0 min (20% B). The flow rate was maintained at 0.6 mL/min. The temperature of the column was 40 °C and detection was performed at 254 nm.

### Cell Viability Assay

Human osteosarcoma (MG-63) and keratinocyte (HaCaT) cells were seeded in 96-well plates at a density of 4 × 10^4^ cells/mL (100 μL/well) in DMEM supplemented with 10% fetal bovine serum and 1% penicillin/streptomycin. After 24 h of incubation at 37 °C with 5% CO_2_, cells were treated with various concentrations of extracts (TAFE-E, TAFE-H, TAFE-C, TAFE-A, and TAFE-B; 250–3.9 μg/mL), the isolated compound Xylopine (30–0.46 μg/mL), or doxorubicin (20–0.3 μg/mL, positive control). Untreated cells served as the negative control. Following a 48 h treatment, MTT solution (0.4 mg/mL) was added and incubated for 4 h. Formazan crystals were dissolved in dimethyl sulfoxide (DMSO), and absorbance was measured at 570 nm.^13^

### Calculation of the Selectivity Index (SI)

The selectivity index of the tested samples and the positive control was calculated by dividing the IC_50_ of HaCat cells by the IC_50_ of MG-63 cells. Samples with anticancer potential are considered selective against cancer cells; those with a selectivity index ≥2.

### Bacterial Strains

Strains were obtained from the Laboratory of Gram-Positive Cocci of Fluminense Federal University, Brazil. Bacterial strains *S. aureus* ATCC 29213 and *E. coli* ATCC 25922 were stored in Brain Heart Infusion (BHI) broth with 10% glycerol at −80 °C.

### Minimum Inhibitory Concentration

Bacterial strains were streaked onto Mueller–Hinton agar (MHA) plates, and isolated colonies were suspended in 0.85% NaCl solution. The bacterial suspension was adjusted to 10^8^ colony-forming units (cfu)/mL, corresponding to the 0.5 McFarland standard. Extracts of *T. axilliflora* and xylopine were solubilized in dimethyl sulfoxide (DMSO) at a final concentration of 5% and serially diluted in 96-well microplates containing Mueller–Hinton broth (MHB) to yield final concentrations ranging from 2.000 μg/mL to 15.6 μg/mL. The bacterial inoculum was added to each well to reach a final concentration of 10^5^ cfu/mL.^25^ Vancomycin and ciprofloxacin were used as positive controls for *S. aureus* and *E. coli*, respectively, while 5% DMSO was employed as the negative control. After incubation at 37 °C for 24 h, 20 μL of resazurin solution was added to each well to determine the MIC.^26^ Tests were performed in triplicate following the standard broth microdilution method described by CLSI (2023).^25^

### Minimum Bactericidal Concentration

Following MIC incubation, 10 μL of each well sample was applied to a Petri plate with MHA, corresponding to each concentration of the microdilution, including positive controls. The plates were then incubated for 24 h at 37 °C. The MBC end point is defined as the last visible bacterial growth, indicating elimination of 99.9% of bacteria.^26^

### Animals

The experimental protocols used in this study were approved by the Ethics Committee for the Care and Use of Experimental Animals of Rio de Janeiro State University (CEUA UERJ 032/2025). Male Swiss mice (*Mus musculus*), weighing between 25 and 30 g and age-matched (between 5 and 6 weeks) were obtained and housed at the animal facility of the Department of Pharmacology and Psychobiology (DFP) at the Instituto de Biologia Roberto Alcantara Gomes (IBRAG) of the State University of Rio de Janeiro (UERJ). Animals were housed under controlled conditions (12 h light/dark cycle, lights off 6:00 p.m.–6:00 a.m.; 21 ± 1 °C; 50 ± 2% relative humidity) in polypropylene cages with sawdust bedding, four per cage. Food (standard pellet diet) and water were available ad libitum. Prior to behavioral testing, mice were acclimated to the experimental room for 30 min and randomly assigned to groups. Drugs were freshly dissolved in DMSO immediately before administration and were coded by an independent experimenter. Injections were performed by one investigator, and behavioral scoring by another blinded to allocation. Codes were revealed only after data analysis.

### Formalin Test

The formalin test is a biphasic acute pain model, characterized by an initial phase or neurogenic phase (0–5 min), a brief quiescent period, and a second phase or inflammatory phase (15–45 min).^27,28^ Formalin (20 μL, 2.5%) was administered via intraplantar injection into the right hind paw of male mice (*n* = 7 per group), 30 min after intraperitoneal (i.p.) treatment with vehicle (DMSO), morphine, acetylsalicylic acid, ethanolic extract of TAFE, or xylopine. DMSO served as the negative control, and morphine and acetylsalicylic acid were used as positive controls for the neurogenic and inflammatory phases, respectively. Immediately afterward, the animals were placed in a plexiglass chamber, and nociceptive behavior—measured as the time spent licking the injected paw—was recorded over a 45 min period.

### Computational Prediction of Physicochemical Descriptors (SwissADME)

The critical descriptors encompassed by Lipinski’s rule of five: molecular weight (*M*_r_ [g/mol]), number of hydrogen bond acceptors (nHBA), number of hydrogen bond donors (nHBD), and the logarithm of the partition coefficient (logP), were assessed using the SwissADME web-based platform (http://www.swissadme.ch). This freely accessible resource integrates a suite of robust and high-performance computational models for predicting physicochemical parameters, pharmacokinetic behavior, and drug-likeness of small organic compounds.

### Statistical Analysis

Statistical analyses were carried out using GraphPad Prism version 8.0. For cytotoxicity assays, three biological and three technical triplicates were performed, considering the control group (CTRL) as 100% cell viability. Antinociceptive data were expressed as mean ± standard error of the mean (SEM), and microbiological results were obtained from three independent experiments. All data sets were evaluated by one-way ANOVA, and when appropriate, Dunnett’s post hoc test was applied for multiple comparisons.

## Supplementary Material



## Notes

The NMR, HRMS, and HPLC-UV data for the extract and xylopine isolated and purified are available. In silico data of xylopine are provided (PDF)
