# Supplementary material for: Bioactivities and Phytochemical Profiling of Trigynaea axilliflora from Brazil
Source: ACS Omega. 2025 Nov 19;10(47):57630–40. doi: 10.1021/acsomega.5c08985 (PMC12676495; doi:10.1021/acsomega.5c08985)
Supplement: Supplementary file 1 [file ao5c08985_si_001.pdf]

SUPPORTING INFORMATION

Bioactivities and phytochemical profiling of *Trigynaea axilliflora* from Brazil

Natyele Rodrigues Fonseca[a], Diogo Folly Gomes Andrade[b], Julia Chaves Scaffo[b,e], Bismarck Rezende[c], Vitória Macario de Simas Gonçalves[c], Yure Bazilio dos Santos[c], Maria Eduarda Barros de Andrade[c], Karine Simões Calumbi[c], Milena Longue[d], Bruno de Araújo Penna[e], Adriana Quintella Lobão[f], Leandro Machado Rocha[b], Alessandra Leda Valverde[a], Guilherme Carneiro Montes[c], Aislan Cristina Rheder Fagundes Pascoal[d] and Lucas Silva Abreu[a]*

[a] Instituto de Química, Universidade Federal Fluminense, 24020-141, Niterói - RJ, Brazil

[b] Faculdade de Farmácia, Universidade Federal Fluminense, 24241-000, Niterói - RJ, Brazil

[c] Departamento de Farmacologia e Psicobiologia, Instituto de Biologia Roberto Alcantara Gomes, Universidade do Estado do Rio de Janeiro, 20551-030, Rio de Janeiro - RJ, Brazil

[d] Instituto de Saúde de Nova Friburgo, Universidade Federal Fluminense, 28625-650, Nova Friburgo - RJ, Brazil

[e] Instituto Biomédico, Universidade Federal Fluminense, 24210-130, Niterói - RJ, Brazil

[f] Instituto de Biologia, Departamento de Biologia Geral, Universidade Federal Fluminense, 24210-201, Niterói - RJ, Brazil

Table of contents

Title	Page
**Figure S1.** ^{1}H NMR spectrum of xylopine obtained from fraction C8 from VLC (500MHz, MeOD)	**3**
**Figure S2.** ^{13}C NMR spectrum of xylopine (125 MHz, MeOD)	**3**
**Figure S3.** HSQC spectrum of xylopine (500/125 MHz, MeOD)	**4**
**Figure S4.** HMBC spectrum of xylopine (500/125 MHz, MeOD)	**4**
**Figure S5.** COSY spectrum of xylopine (500/125 MHz, MeOD)	**5**
**Figure S6.** HPLC-UV chromatogram for xylopine obtained from VLC	**5**
**Figure S7.** ^{1}H NMR spectrum of xylopine obtained from method 1 (500MHz, MeOD)	**6**
**Figure S8.** ^{1}H NMR spectrum of xylopine obtained from method 2 (500MHz, MeOD)	**6**
**Figure S9.** ^{1}H NMR spectrum of xylopine obtained from method 3 (500MHz, MeOD)	**7**
**Figure S10.** HPLC-DAD chromatogram in 254 nm of xylopine obtained from method 1 and data of purity.	**8**
**Figure S11.** HPLC-DAD chromatogram in 254 nm of xylopine obtained from method 2 and data of purity.	**9**
**Figure S12.** HPLC-DAD chromatogram in 254 nm of xylopine obtained from method 3 (acid-base) and data of purity.	**10**
**Figure S13.** HPLC-DAD chromatogram in 254 nm of crude ethanolic extract and data of purity of xylopine.	**11**
**Table S1.** ^{1}H and ^{13}C NMR data of Xylopine	**12**
**Table S2.** Characterization of the compounds tentatively identified by HPLC-ESI-MS from the leaves of ethanolic extract of *T. axilliflora*	**13**
**Table S3.** Physicochemical Properties of Xylopine	**13**
**Table S4.** Water Solubility of Xylopine.	**14**
**Table S5.** Lipophilicity of Xylopine.	**14**
**Table S6.** Pharmacokinetics	**14**
**Table S7.** Druglikeness and Medicinal Chemistry	**15**

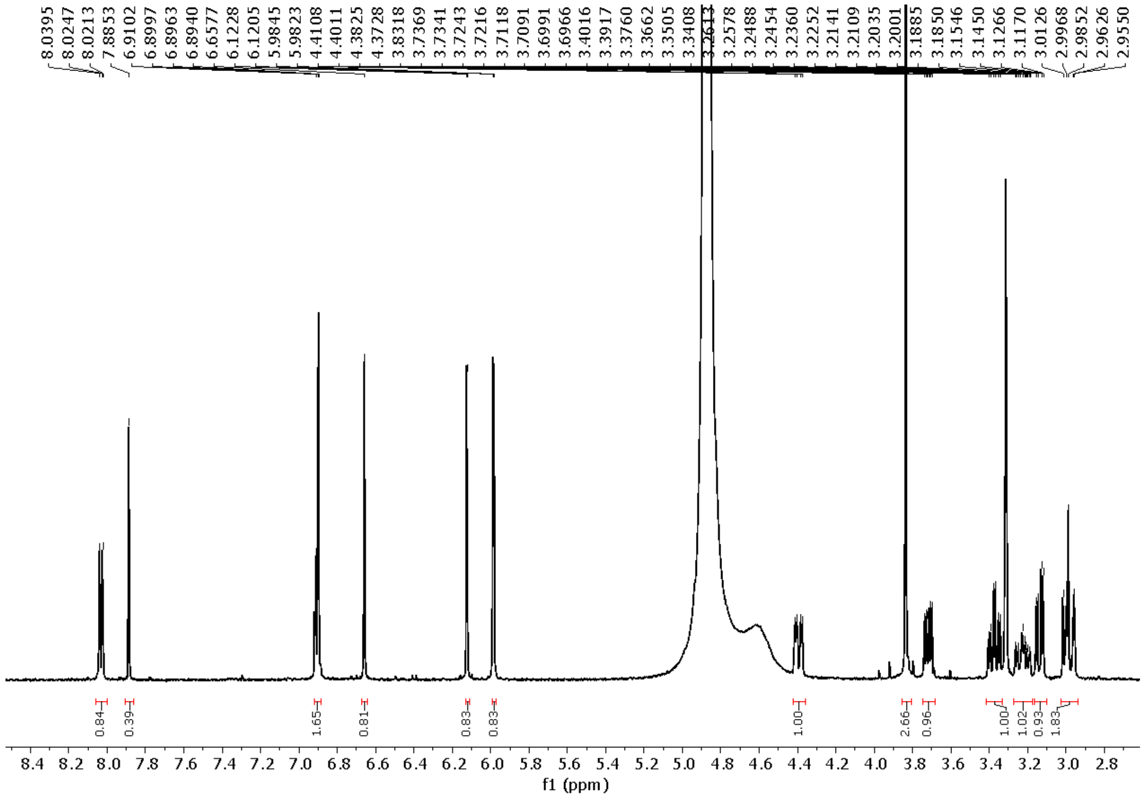

Figure S1. ^{1}H NMR spectrum of xylopine obtained from fraction C8 from VLC (500MHz, MeOD).

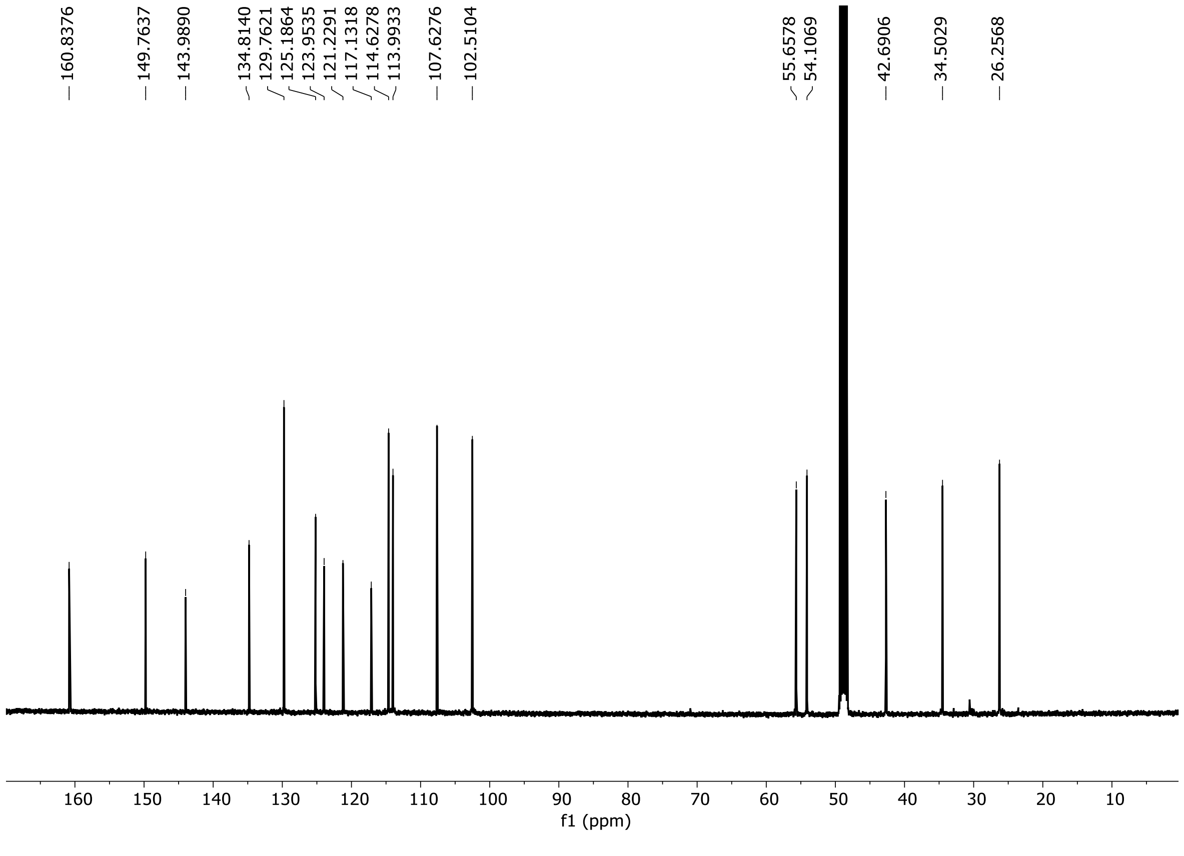

Figure S2. ^{13}C NMR spectrum of xylopine (125 MHz, MeOD).

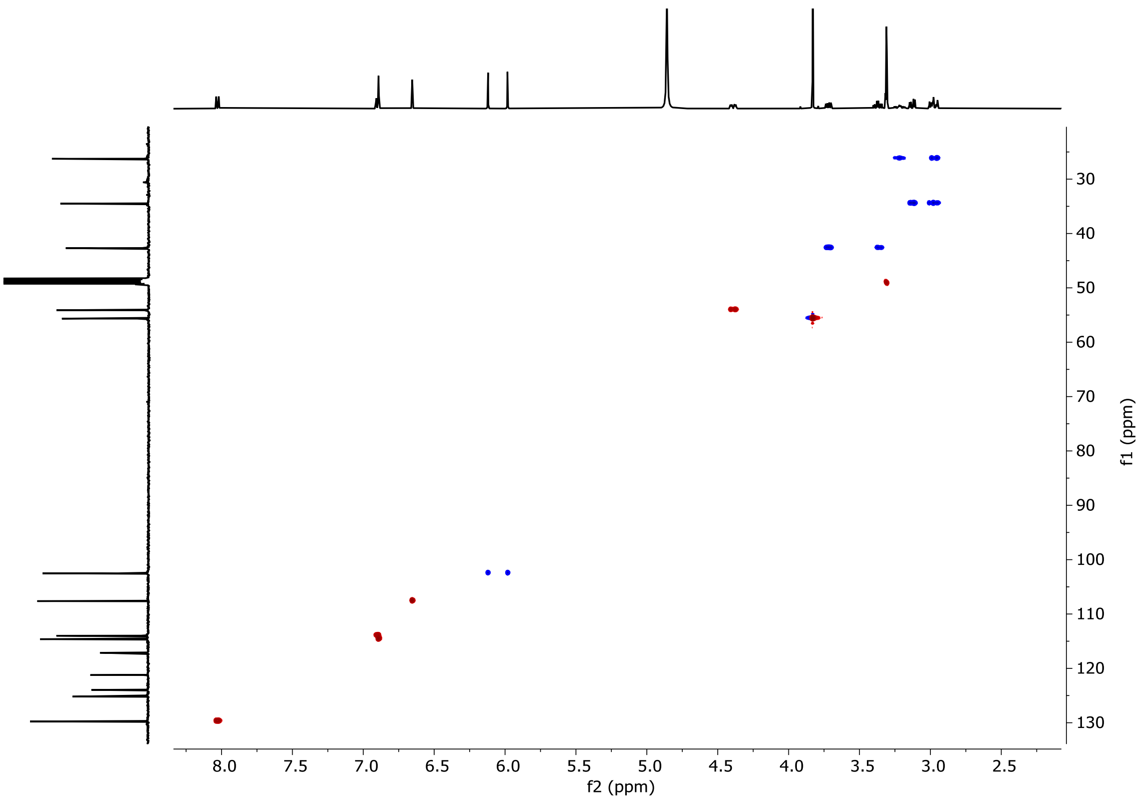

Figure S3. HSQC spectrum of xylopine (500/125 MHz, MeOD).

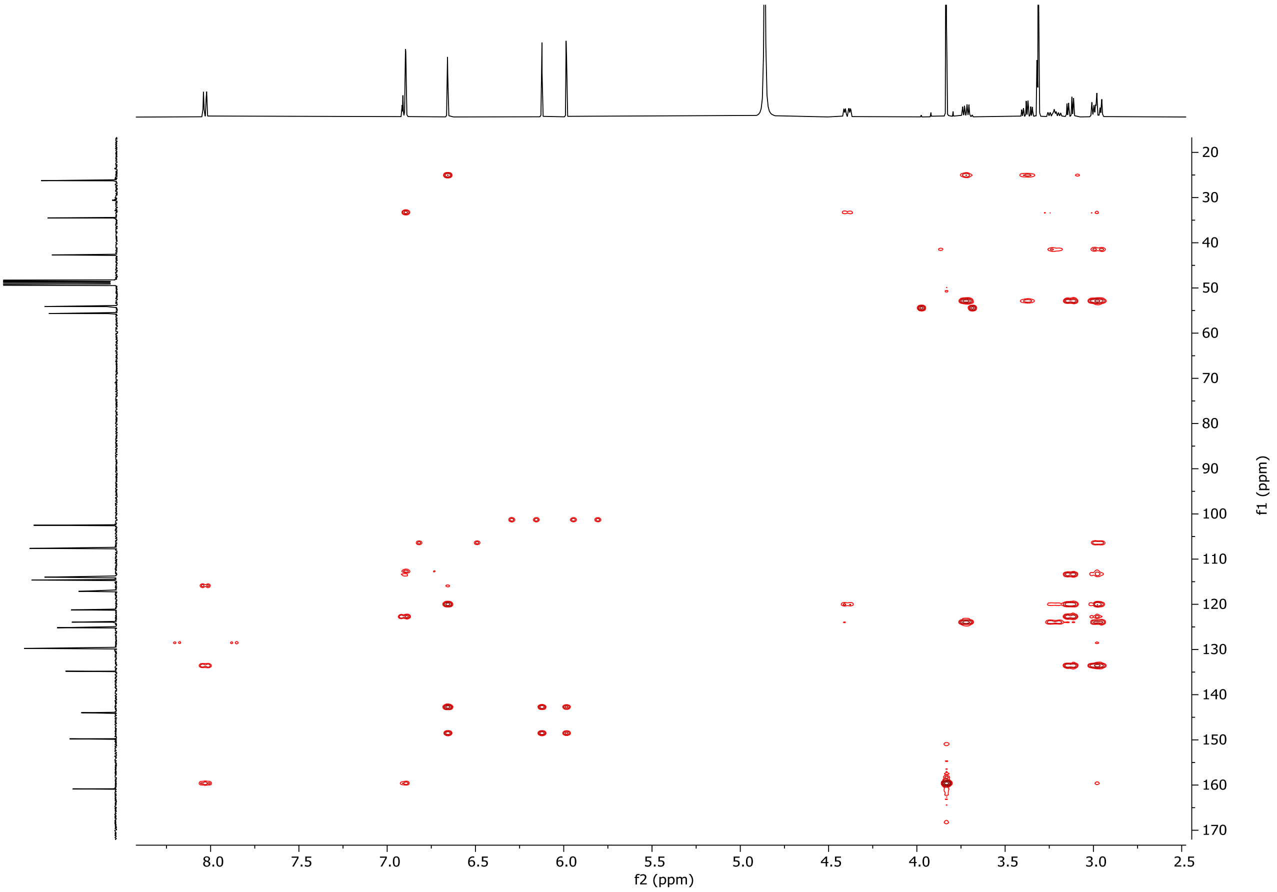

Figure S4. HMBC spectrum of xylopine (500/125 MHz, MeOD).

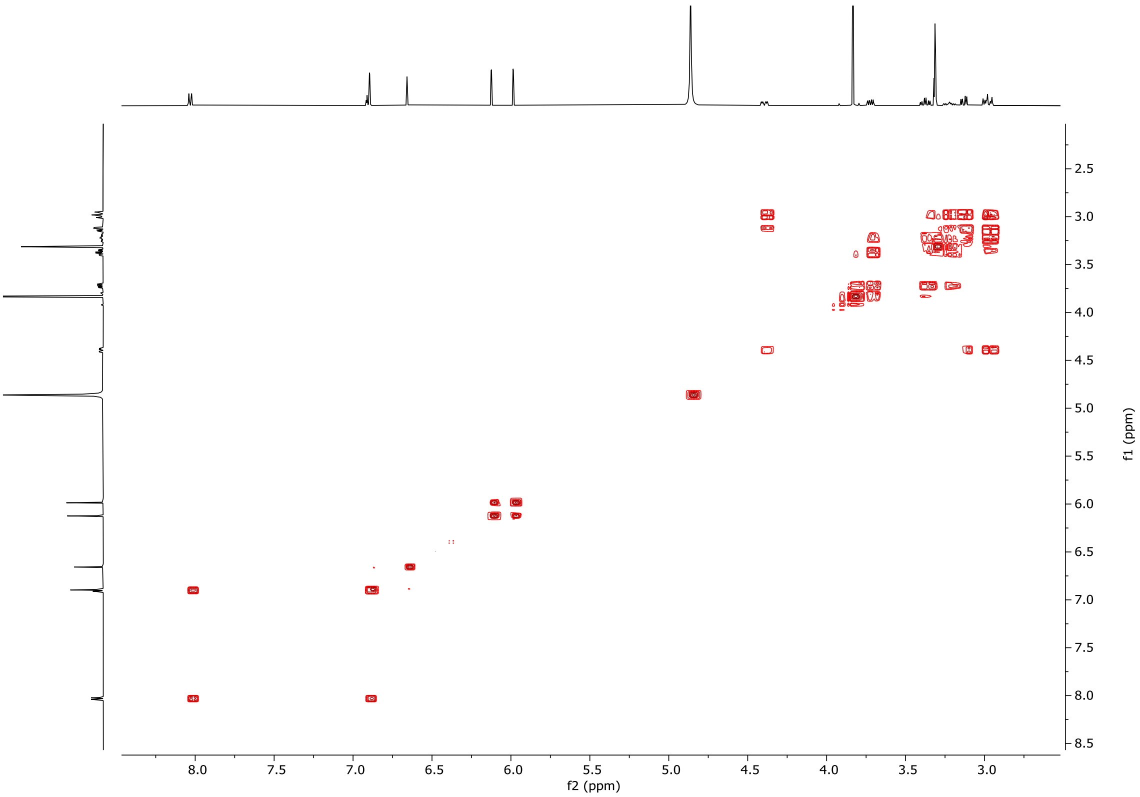

Figure S5. COSY spectrum of xylopine (500/125 MHz, MeOD).

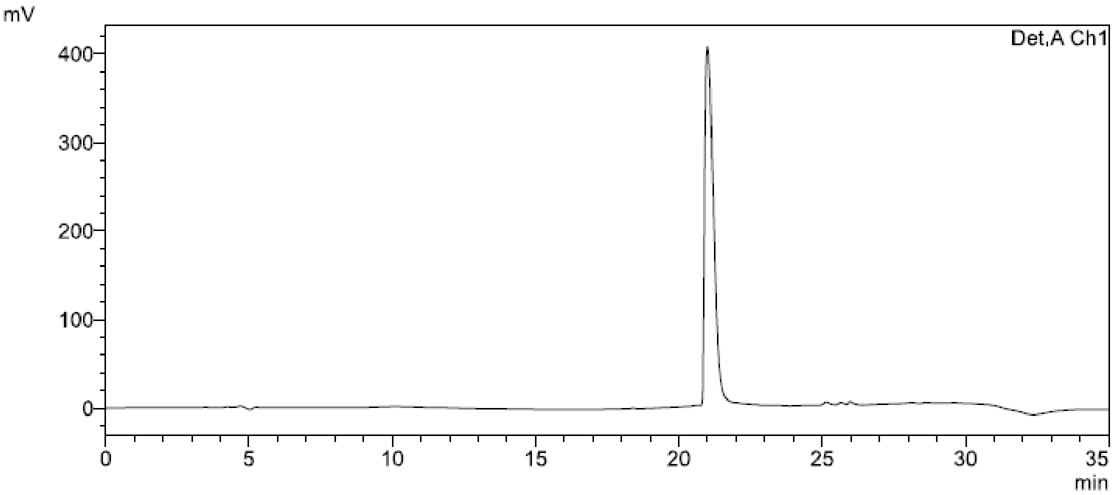

Figure S6. HPLC-UV chromatogram in 254 nm of xylopine obtained from VLC.

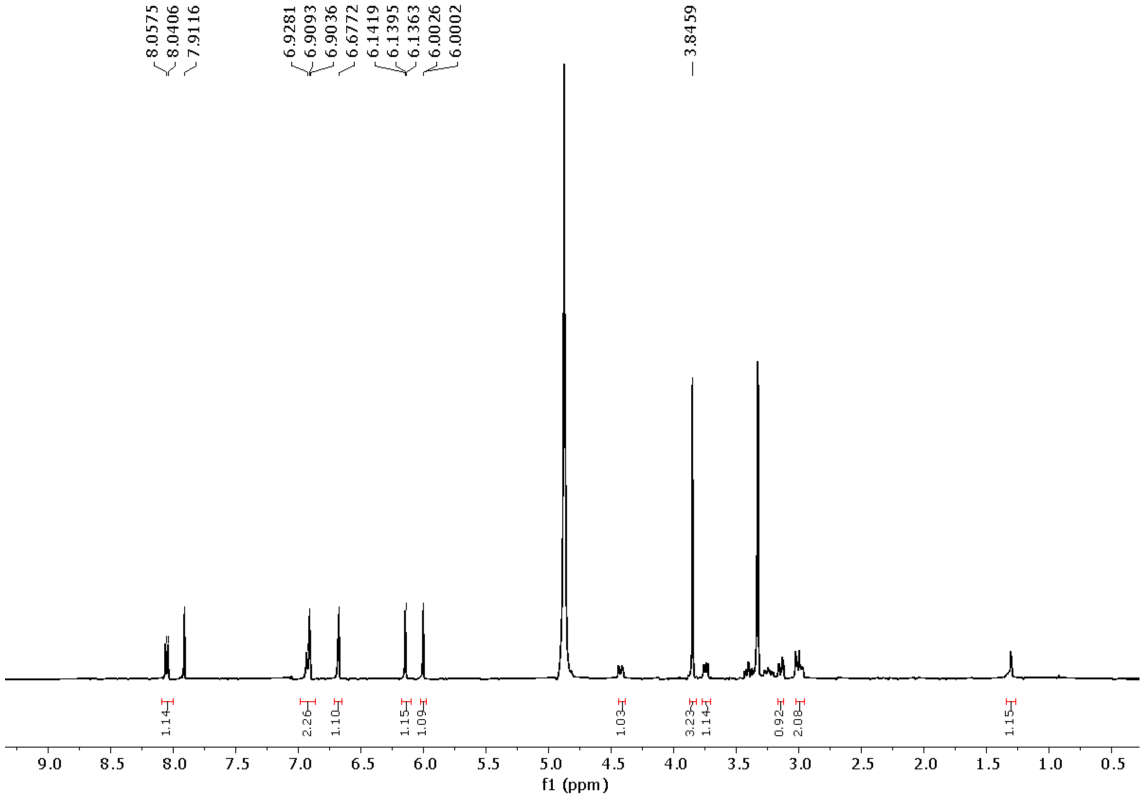

Figure S7. ¹H NMR spectrum of xylopine obtained from method 1 (500MHz, MeOD).

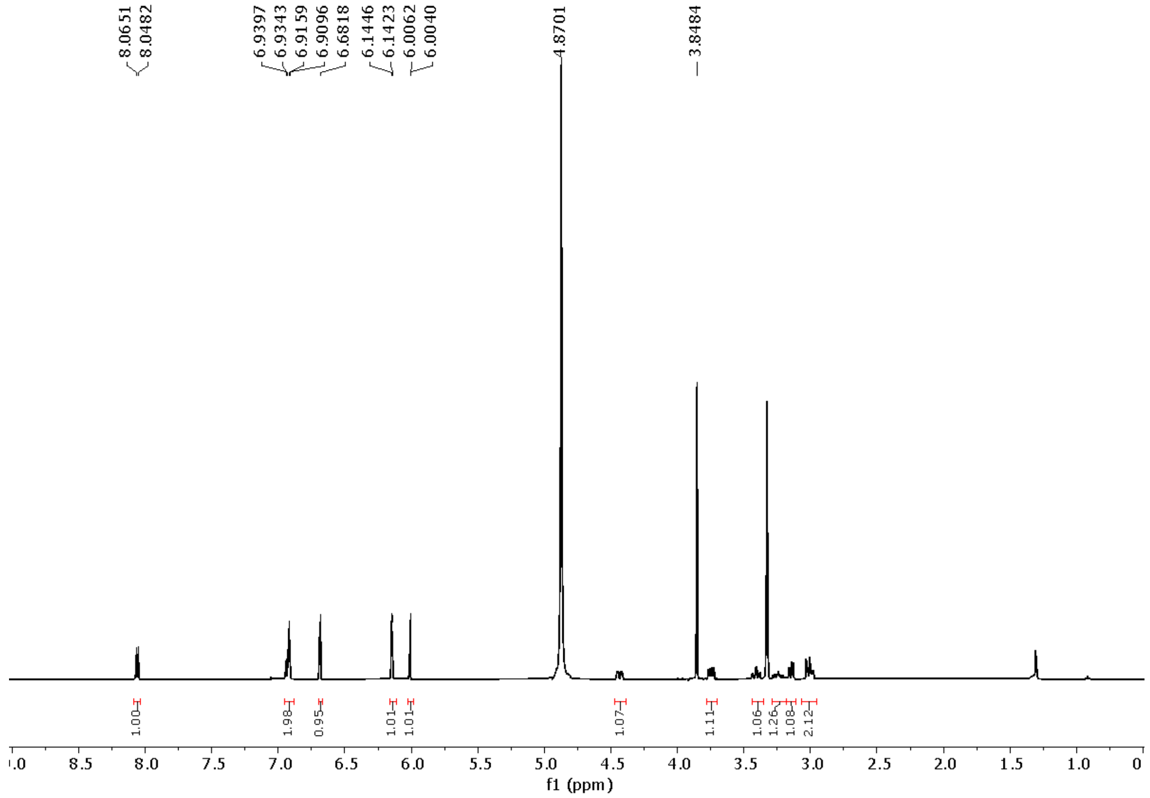

Figure S8. ¹H NMR spectrum of xylopine obtained from method 2 (500MHz, MeOD).

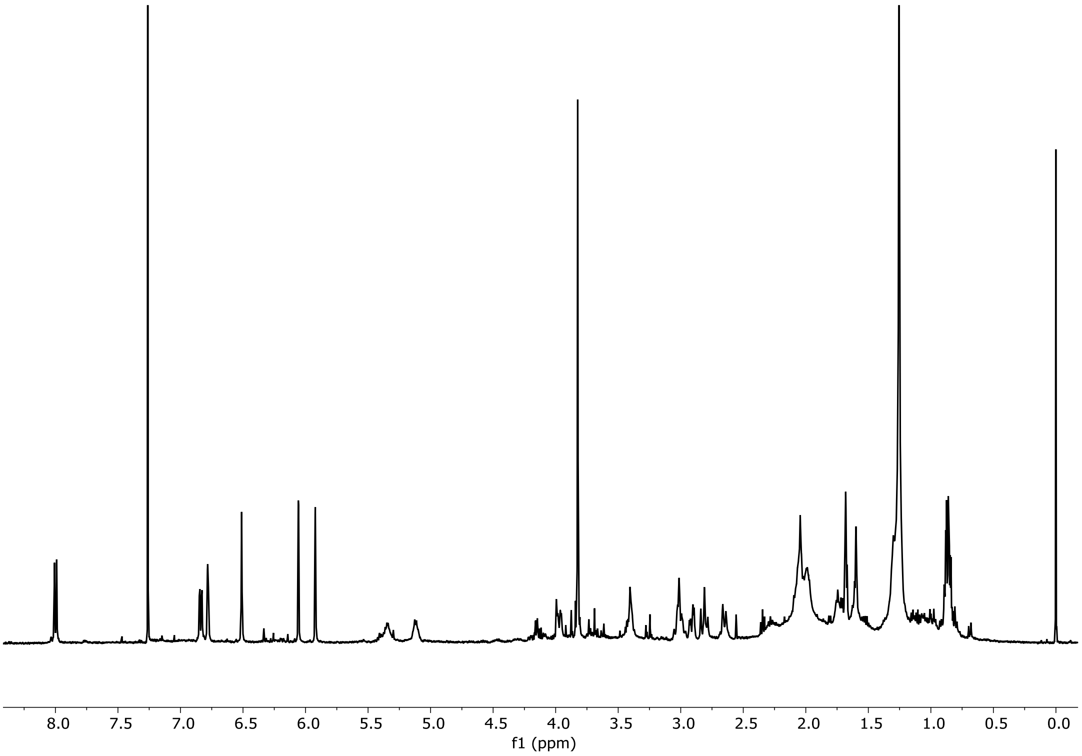

Figure S9. ^{1}H NMR spectrum of xylopine obtained from method 3 (acid-base) (500MHz, MeOD).

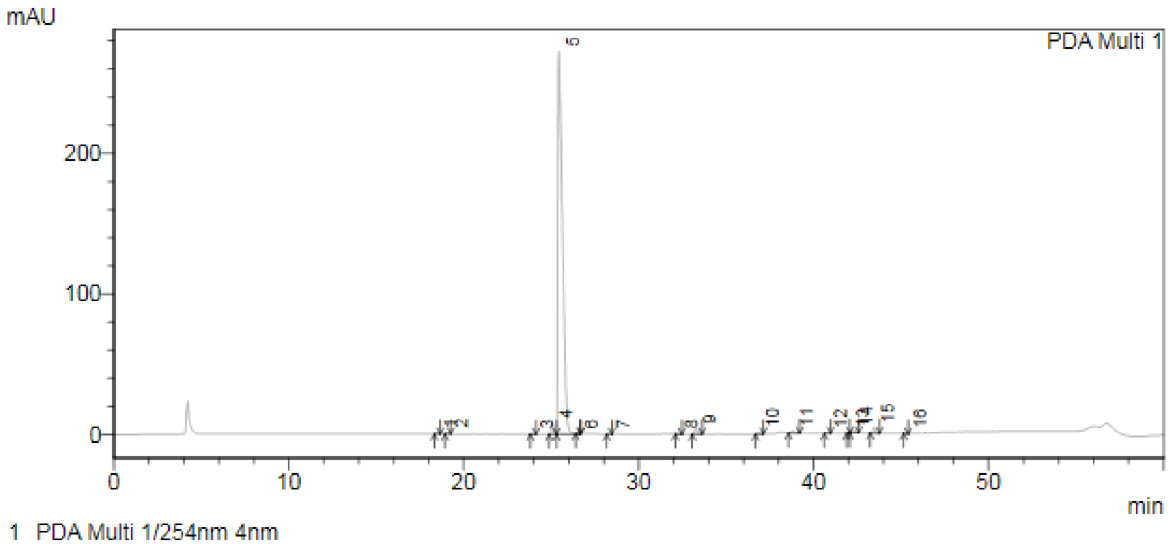

PeakTable

PDA Ch1 254nm 4nm

Peak#	Ret. Time	Area	Height	Area %	Height %
1	18.464	6243	861	0.122	0.290
2	19.073	6688	945	0.131	0.318
3	23.942	3258	410	0.064	0.138
4	25.049	58820	7693	1.153	2.590
5	25.437	4873253	272178	95.542	91.626
6	26.489	2100	321	0.041	0.108
7	28.302	2593	332	0.051	0.112
8	32.212	2891	351	0.057	0.118
9	33.258	40826	3969	0.800	1.336
10	36.848	13487	1285	0.264	0.432
11	38.802	18471	1159	0.362	0.390
12	40.748	3567	401	0.070	0.135
13	42.016	5103	759	0.100	0.256
14	42.209	29994	1979	0.588	0.666
15	43.433	29548	3858	0.579	1.299
16	45.281	3780	554	0.074	0.186
Total		5100622	297055	100.000	100.000

Figure S10. HPLC-DAD chromatogram in 254 nm of xylopine obtained from method 1 and data of purity.

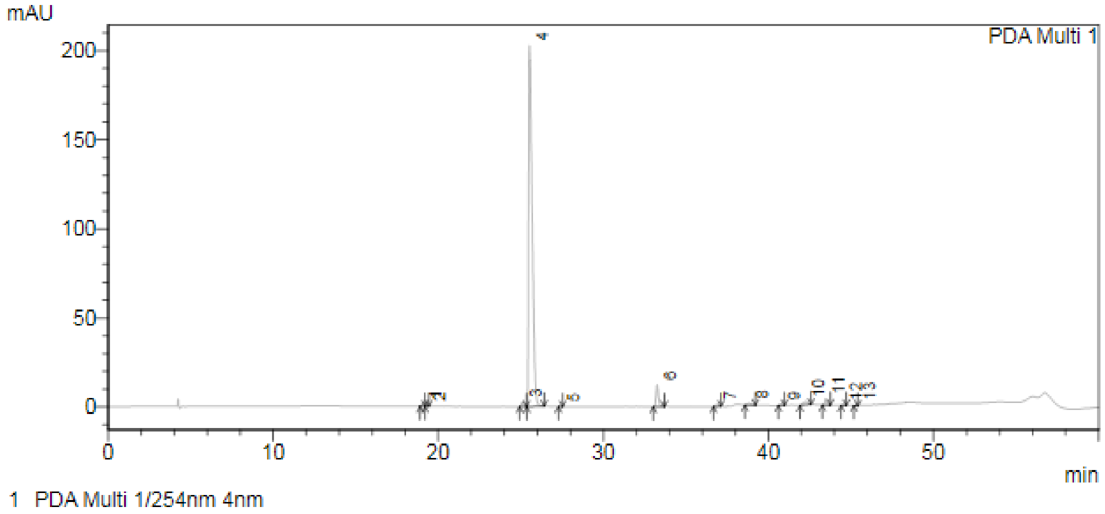

PeakTable

PDA Ch1 254nm 4nm

Peak#	Ret. Time	Area	Height	Area %	Height %
1	19.041	13571	1831	0.408	0.797
2	19.285	2428	307	0.073	0.133
3	25.105	22640	2736	0.681	1.190
4	25.513	3055413	202723	91.842	88.203
5	27.393	1445	211	0.043	0.092
6	33.257	125939	12468	3.786	5.425
7	36.865	13104	1252	0.394	0.545
8	38.821	17987	1140	0.541	0.496
9	40.770	4621	509	0.139	0.221
10	42.226	34045	1932	1.023	0.841
11	43.454	28934	3825	0.870	1.664
12	44.509	2462	280	0.074	0.122
13	45.311	4226	622	0.127	0.270
Total		3326815	229836	100.000	100.000

Figure S11. HPLC-DAD chromatogram in 254 nm of xylopine obtained from method 2 and data of purity.

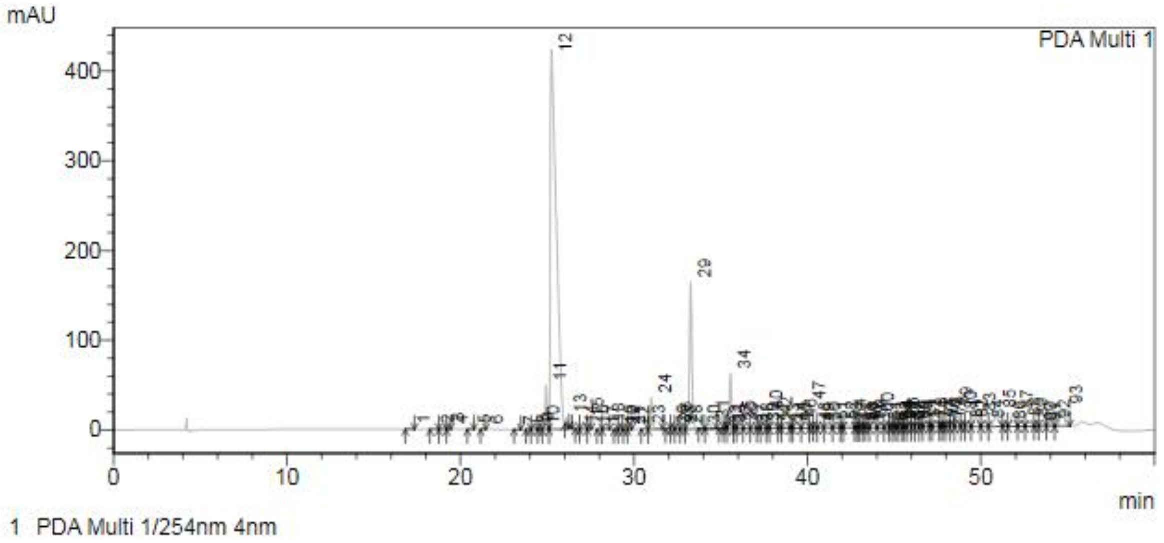

PeakTable

PDA Ch1 254nm 4nm

Peak#	Ret. Time	Area	Height	Area %	Height %
1	17.056	18081	1442	0.100	0.126
2	18.434	13937	1335	0.077	0.117
3	19.041	29555	3404	0.164	0.298
4	19.253	6628	807	0.037	0.071
5	20.571	11449	1370	0.063	0.120
6	21.298	7690	1082	0.043	0.095
7	23.212	3699	291	0.020	0.025
8	23.925	11044	1416	0.061	0.124
9	24.194	38142	3793	0.211	0.332
10	24.545	3561	502	0.020	0.044
11	24.925	395995	48785	2.193	4.275
12	25.236	10106379	423442	55.957	37.104
13	26.093	57140	8702	0.316	0.762
14	26.717	5106	761	0.028	0.067
15	27.104	66689	8670	0.369	0.760
16	27.392	4135	654	0.023	0.057
17	27.997	2569	330	0.014	0.029
18	28.300	25556	3195	0.141	0.280
19	28.992	4774	580	0.026	0.051
20	29.173	3551	410	0.020	0.036
21	29.494	2740	382	0.015	0.033
22	29.736	1840	253	0.010	0.022

Peak#	Ret. Time	Area	Height	Area %	Height %
23	30.622	15631	1183	0.087	0.104
24	30.989	314916	35495	1.744	3.110
25	32.025	9141	887	0.051	0.078
26	32.208	31672	3842	0.175	0.337
27	32.465	17249	1565	0.096	0.137
28	32.778	4519	497	0.025	0.044
29	33.260	1604514	163653	8.884	14.340
30	33.805	2282	333	0.013	0.029
31	34.334	59844	6014	0.331	0.527
32	35.051	1833	268	0.010	0.023
33	35.275	4572	710	0.025	0.062
34	35.554	547831	61679	3.033	5.405
35	35.800	41392	5047	0.229	0.442
36	35.936	29557	3608	0.164	0.316
37	36.593	12250	1021	0.068	0.089
38	36.861	29183	2195	0.162	0.192
39	37.224	20397	2534	0.113	0.222
40	37.409	146672	16264	0.812	1.425
41	37.761	25521	2947	0.141	0.258
42	37.960	124889	8949	0.691	0.784
43	38.389	15834	1511	0.088	0.132
44	38.817	77177	3087	0.427	0.270
45	39.065	26478	2810	0.147	0.246
46	39.387	105137	7000	0.582	0.613
47	39.872	257545	26430	1.426	2.316
48	40.184	34721	3174	0.192	0.278
49	40.411	25265	2082	0.140	0.182
50	40.756	46685	3205	0.258	0.281

50	40.756	46685	3205	0.258	0.281
51	41.192	71551	3591	0.396	0.315
52	41.744	60274	3434	0.334	0.301
53	42.016	27390	2930	0.152	0.257
54	42.228	139583	6490	0.773	0.569
55	42.795	16324	2016	0.090	0.177
56	42.949	21723	2660	0.120	0.233
57	43.115	31768	3417	0.176	0.299
58	43.254	31527	3473	0.175	0.304
59	43.434	57632	5689	0.319	0.498
60	43.823	150594	13174	0.834	1.154
61	44.227	61254	3270	0.339	0.287
62	44.604	42351	2615	0.234	0.229
63	44.832	30945	3137	0.171	0.275
64	44.966	43379	4832	0.240	0.423
65	45.131	31254	3989	0.173	0.349
66	45.281	61197	7240	0.339	0.634
67	45.493	26464	2888	0.147	0.253
68	45.639	29978	3327	0.166	0.292
69	45.872	54185	4073	0.300	0.357
70	46.077	41366	3817	0.229	0.334
71	46.277	69657	5368	0.386	0.470
72	46.519	66221	5803	0.367	0.508
73	46.891	100354	6581	0.556	0.577
74	47.077	39098	4138	0.216	0.363
75	47.456	140586	7135	0.778	0.625
76	47.691	47361	5490	0.262	0.481
77	47.816	60320	5722	0.334	0.501
78	48.122	99614	8387	0.552	0.735
79	48.336	209324	18079	1.159	1.584
80	48.590	201749	14115	1.117	1.237
81	48.949	59738	4860	0.331	0.426
82	49.231	84040	4957	0.465	0.434
83	49.699	199821	12066	1.106	1.057
84	50.173	85577	4113	0.474	0.360
85	50.848	276056	15443	1.528	1.353
86	51.420	42874	2841	0.237	0.249
87	51.858	231793	14692	1.283	1.287
88	52.280	92972	6082	0.515	0.533
89	52.718	85666	5237	0.474	0.459
90	53.195	11371	805	0.063	0.071
91	53.447	23534	1125	0.130	0.099
92	53.989	44005	3209	0.244	0.281
93	54.681	305631	19320	1.692	1.693
Total		18061062	1141226	100.000	100.000

Figure S12. HPLC-DAD chromatogram in 254 nm of xylopine obtained from method 3 (acid-base) and data of purity.

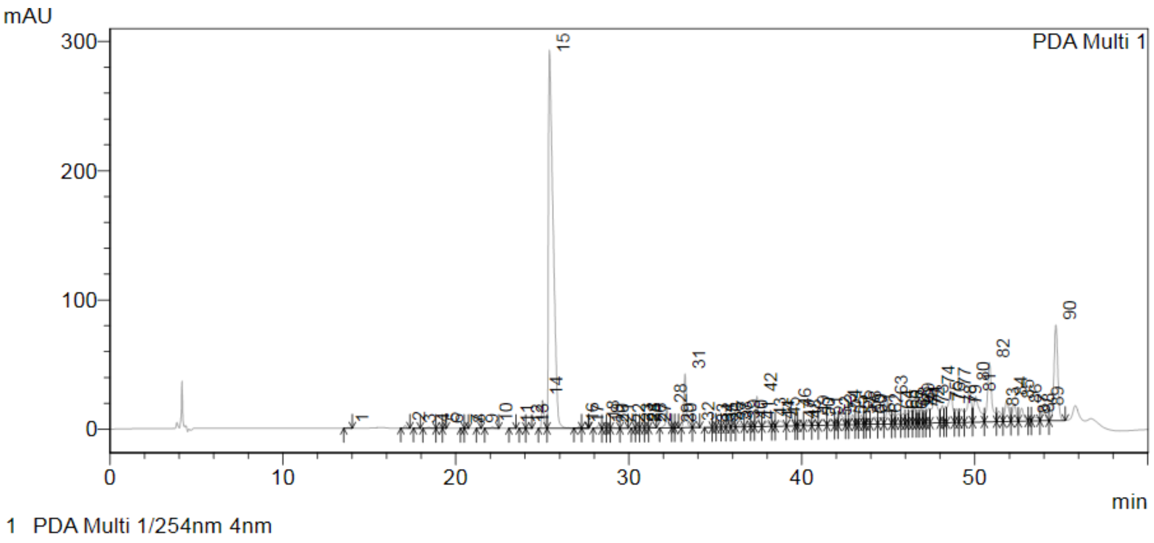

PeakTable

PDA Ch1 254nm 4nm

Peak#	Ret. Time	Area	Height	Area %	Height %
1	13.776	5738	501	0.038	0.052
2	17.068	19014	2150	0.127	0.223
3	17.689	1386	163	0.009	0.017
4	18.443	16829	874	0.112	0.091
5	19.056	11656	1297	0.078	0.134
6	19.275	1389	208	0.009	0.022
7	20.416	1538	174	0.010	0.018
8	20.560	2175	280	0.015	0.029
9	21.293	1994	298	0.013	0.031
10	22.103	17135	1293	0.115	0.134
11	23.314	2051	164	0.014	0.017
12	23.904	2666	233	0.018	0.024
13	24.193	4268	406	0.029	0.042
14	25.008	188751	21826	1.261	2.261
15	25.408	5506671	292823	36.797	30.339
16	27.106	11454	1157	0.077	0.120
17	27.382	3620	371	0.024	0.038
18	28.281	32167	3021	0.215	0.313
19	28.543	3590	385	0.024	0.040
20	28.825	2606	285	0.017	0.029
21	29.097	11181	784	0.075	0.081
22	29.760	6625	661	0.044	0.069
23	30.256	7789	1031	0.052	0.107
24	30.586	7330	862	0.049	0.089
25	30.693	10495	907	0.070	0.094
26	31.010	5525	603	0.037	0.063
27	31.274	11424	813	0.076	0.084
28	32.190	144543	15560	0.966	1.612
29	32.589	1793	227	0.012	0.024
30	32.752	3814	455	0.025	0.047
31	33.245	416090	41874	2.780	4.339
32	33.805	15292	1461	0.102	0.151
33	34.627	3909	228	0.026	0.024
34	34.937	1058	132	0.007	0.014
35	35.189	3765	315	0.025	0.033
36	35.438	7550	895	0.050	0.093
37	35.794	17496	1971	0.117	0.204
38	35.954	11233	1294	0.075	0.134
39	36.424	23084	2431	0.154	0.252
40	36.859	25472	2174	0.170	0.225
41	37.211	16103	1914	0.108	0.198
42	37.401	204546	23655	1.367	2.451
43	37.952	69741	3687	0.466	0.382
44	38.389	21111	1801	0.141	0.187
45	38.743	98029	3986	0.655	0.413
46	39.384	134873	10739	0.901	1.113
47	39.691	13076	1597	0.087	0.165
48	39.955	34088	1832	0.228	0.190
49	40.440	66882	4574	0.447	0.474
50	40.743	51896	2886	0.347	0.299
51	41.190	84639	4024	0.566	0.417
52	41.727	79351	3685	0.530	0.382
53	42.022	52998	5727	0.354	0.593
54	42.227	156989	9002	1.049	0.933
55	42.626	44378	4168	0.297	0.432
56	42.927	124131	7939	0.829	0.823
57	43.144	47525	4575	0.318	0.474
58	43.425	95247	7470	0.636	0.774
59	43.617	42360	4869	0.283	0.504
60	43.773	60063	4639	0.401	0.481
61	44.226	123595	5440	0.826	0.564
62	44.618	105329	5140	0.704	0.532
63	44.959	239438	18996	1.600	1.968
64	45.274	84799	7886	0.567	0.817
65	45.580	128535	7530	0.859	0.780
66	45.854	108227	7765	0.723	0.804
67	46.112	82878	7473	0.554	0.774
68	46.277	137237	10006	0.917	1.037
69	46.504	137972	12544	0.922	1.300
70	46.695	65546	8049	0.438	0.834
71	46.885	124236	11087	0.830	1.149
72	47.079	94769	9449	0.633	0.979
73	47.327	140588	11446	0.939	1.186
74	47.661	510159	26037	3.409	2.698
75	48.117	147544	13528	0.986	1.402
76	48.298	114918	12693	0.768	1.315
77	48.608	434180	24464	2.901	2.535
78	48.938	139966	10559	0.935	1.094
79	49.233	162743	10444	1.087	1.082
80	49.690	454313	28367	3.036	2.939
81	50.046	471500	19830	3.151	2.055
82	50.842	783294	45960	5.234	4.762
83	51.396	145167	7977	0.970	0.827
84	51.852	266808	16534	1.783	1.713
85	52.277	204585	13671	1.367	1.416
86	52.705	164768	10259	1.101	1.063
87	53.202	21444	1926	0.143	0.200
88	53.450	70464	3009	0.471	0.312
89	53.984	119341	7807	0.797	0.809
90	54.685	1110500	73943	7.421	7.661
Total		14965038	965172	100.000	100.000

Figure S13. HPLC-DAD chromatogram in 254 nm of crude ethanolic extract and data of purity of xylopine.

Table S1. ^{1}H and ^{13}C NMR data of Xylopine

	Xylopine		Literature[1]	
Position	δ_H (mult., J in Hz)	δ_C	δ_H (mult., J in Hz)	δ_C, *type*
1		144.0		144.2 C
2		149.7		144.9 C
3	6.66 (s)	107.6	6.67 (s)	107.8 CH
3a		125.1		125.3 C
4	3.22 (m), 2.97 (m)	26.3	2.99 (m)	26.3 CH_2
5ax	3.37 (m)	42.7	3.20 (m)	42.8 CH_2
5eq	3.71 (m)		3.69 (m)	
6a	4.38 (dd, 14.5, 5.0)	54.1	4.4 (dd, 14.2)	54.2 CH
7ax	2.98 (m)	34.5		34.6 CH_2
7eq	3.13 (dd, 14.0, 5.0)		3.10 (dd, 5)	
8	6.89 (d, 1.0)	114.6	6.92 (s)	114.8 CH
9		160.8		161.0 C
10	6.91 (dd, 7.1, 1.0)	114.0	6.94 (d, 7.0)	114.1 CH
11	8.02 (d, 7.1)	129.7	8.05 (d, 7.0)	129.9 CH
11a		124.0		124.1 CH
11b		117.1		117.3 C
11c		121.2		121.2 C
OCH_2O	5.98 (d, 1.1)<br>6.12 (d, 1.1)	102.5	6.01 (d, 1.0);<br>6.13 (d,1,0)	102.7 CH_2
9-OCH_3	3.82 (s)	55.7	3.83 (s)	55.8 CH_3

Table S2. Characterization of the compounds tentatively identified by HPLC-HRESIMS from the leaves of ethanolic extract of *T. axilliflora*

Compound	t_R	*m/z* [M + H]$^+$	Molecular formula	MS/MS	Annotation	Reference
1	10.6	342.1727	$C_{20}H_{24}NO_4$	297; 265; 237; 222	Magnoflorine	2
2	11.2	342.1732	$C_{20}H_{24}NO_4$	297; 265; 237; 222	Laurifoline	2
3	11.4	314.1781	$C_{19}H_{24}NO3$	269; 237; 209; 192; 175	Oblongine	3
4	12.0	312.1225	$C_{18}H_{17}NO_4$	295; 265; 237; 222	Launobine or Actinodaphine	4
5	12.3	342.1727	$C_{20}H_{23}NO_4$	311; 296; 281; 265; 253	Isocorydine	3
6	12.5	356.1883	$C_{21}H_{26}NO_4$	311; 296; 281; 265; 253	Menisperine	3
7	13.1	326.1386	$C_{19}H_{20}NO_4$	295; 265; 237; 222	Bulbocapnine	4
8	15.1	296.1293	$C_{18}H_{17}NO_3$	279; 264; 249; 221; 206; 178	Xylopine	4

Table S3. Physicochemical Properties of Xylopine.

Property	Value
SMILES	COc1ccc2c(c1)C(=O)N(C)[C@H]3c2c2OC(=O)C2C3CN1
Formula	C18H17NO3
Molecular weight	295.33 g/mol
No. of heavy atoms	22
No. of aromatic heavy atoms	12
Fraction Csp3	0.33
No. of rotatable bonds	1
No. of H-bond acceptors	4
No. of H-bond donors	1
Molar refractivity	88.63
TPSA	39.72 Å^2

Table S4. Water Solubility of Xylopine.

Method		Solubility (mg/ml; mol/l)	Class
Log S (ESOL)	-3.77	4.88e-02; 1.98e-04	Soluble
Log S (Ali)	-3.29	1.51e-01; 5.12e-04	Soluble
Log S (SILICOS-IT)	-5.71	5.77e-04; 1.95e-06	Moderately soluble

Table S5. Lipophilicity of Xylopine.

Method

Property	Result
Log P $_{o/w}$ (iLOGP)	3.12
Log P $_{o/w}$ (XLOGP3)	2.80
Log P $_{o/w}$ (WLOGP)	2.13
Log P $_{o/w}$ (MLOGP)	2.49
Log P $_{o/w}$ (SILICOS-IT)	3.84
Consensus Log P $_{o/w}$	2.88

Table S6. Pharmacokinetics

GI absorption	High
BBB permeant	Yes
P-gp substrate	Yes
CYP1A2 inhibitor	Yes
CYP2C19 inhibitor	Yes
CYP2C9 inhibitor	No
CYP2D6 inhibitor	Yes
CYP3A4 inhibitor	Yes

Table S7. Druglikeness and Medicinal Chemistry

Lipinski	Sim; 0 violation
Ghose	Yes
Veber	Yes
Egan	Yes
Muegge	Yes
Bioavailability Score	0.55
PAINS	0 alerta
Brenk	0 alerta
Leadlikeness	Yes
Synthetic accessibility	3.50

REFERENCES

[1] Silva, M. S.; Tavares, J. F.; Queiroga, K. F.; Agra, M. F.; Barbosa-Filho, J. M.; Almeida, J. R. G. S.; Silva, S. A. S. Alcaloides e Outros Constituintes de *Xylopia langsdorffiana* (Annonaceae). *Quim. Nova* **2009**, *32 (6),* 1566–1570.

[2] Sim, H.-J.; Kim, J. H.; Lee, K. R.; Hong, J. Simultaneous Determination of Structurally Diverse Compounds in Different Fangchi Species by UHPLC-DAD and UHPLC-ESI-MS/MS. *Molecules* **2013**, *18* (5), 5235–5250.

[3] Singh, A.; Bajpai, V.; Sharma, K. R.; Kumar, B. Analysis of Isoquinoline Alkaloids from *Mahonia leschenaultia* and *Mahonia napaulensis* Roots Using UHPLC-Orbitrap-MSn and UHPLC-QqQLIT-MS/MS. *J. Pharm. Anal* **2017**, *7 (2*), 77-86.

[4] Mohammed, M. A.; Ashour, R. M.; Alseekh, S.; Farag, M. A. Comprehensive Tools of Alkaloid/Volatile Compounds–Metabolomics and DNA Profiles: Bioassay-Role-Guided Differentiation Process of Six *Annona* sp. Grown in Egypt as Anticancer *Therapy. Pharmaceuticals* **2024**, *17* (1), 103.
